# Design, synthesis and biological evaluation of tricyclic pyrazolo[1,5-*c*][1,3]benzoxazin-5(5*H*)-one scaffolds as selective BuChE inhibitors

**DOI:** 10.1080/14756366.2018.1488696

**Published:** 2018-10-04

**Authors:** Guo-Liang Qiu, Shao-Sheng He, Shi-Chao Chen, Bo Li, Hui-Hui Wu, Jing Zhang, Wen-Jian Tang

**Affiliations:** a School of Pharmacy, Anhui Medical University, Hefei, PR China;; b Lujiang County People's Hospital, Lujiang, Anhui, PR China;; c Anhui Prevention and Treatment Center for Occupational Disease, Hefei, PR China

**Keywords:** acetylcholinesterase, butyrylcholinesterase, pyrazolo[1,5-*c*][1,3]benzoxazin-5(5*H*)-one, tricyclic scaffold, Alzheimer’s disease

## Abstract

Based on the structural analysis of tricyclic scaffolds as butyrylcholinesterase (BuChE) inhibitors, a series of pyrazolo[1,5-*c*][1,3]benzoxazin-5(5*H*)-one derivatives were designed, synthesized and evaluated for their acetylcholinesterase (AChE) and BuChE inhibitory activity. Compounds with 5-carbonyl and 7- or/and 9-halogen substitutions showed potential BuChE inhibitory activity, among which compounds **6a**, **6c** and **6g** showed the best BuChE inhibition (IC_50_ = 1.06, 1.63 and 1.63 µM, respectively). The structure–activity relationship showed that the 5-carbonyl and halogen substituents significantly influenced BuChE activity. Compounds **6a** and **6g** were found nontoxic, lipophilic and exhibited remarkable neuroprotective activity and mixed-type inhibition against BuChE (*K*_i_ = 7.46 and 3.09 µM, respectively). Docking studies revealed that compound **6a** can be accommodated into BuChE via five hydrogen bonds, one Pi–Sigma interaction and three Pi–Alkyl interactions.

## 1. Introduction

Alzheimer’s disease (AD), characterized by progressive deterioration of memory and other cognitive impairments, is a chronic neurodegenerative disease^1^^,^^2^. Forty-seven million people are living with dementia worldwide according to World Alzheimer Report 2016, and this number will increase to more than 131 million by 2050^3^. In the past decades, various pathogenesis hypothesis of AD have been proposed, such as cholinergic dysfunction, β-amyloid oligomerisation, tau-protein hyperphosphorylation and oxidative stress, etc^4–8^. Among them, cholinergic dysfunction hypothesis was the most effective therapeutic strategy. Thus, one efficient approach to treat AD is to restore the level of acetylcholine using cholinesterases (ChEs), such as acetylcholinesterase (AChE) and butyrlcholinesterase (BuChE) inhibitors^9–11^. It has been found that amyloid protein plaques can be caused by ChEs and can be decreased by ChEs inhibitors^12^. Currently, marketed AD drugs are mainly ChEs inhibitors rivastigmine, galantamine and donepezil. Thus, the search for new ChE inhibitors is still of great interest^13–17^.

AChE inhibitors have been used for clinical AD treatments. AChE plays an important role in the hydrolysis of ACh in normal brain, while BuChE takes over the hydrolysis of ACh in the AChE deficient brain^18^^,^^19^. In the hippocampi of AChE deficient mice, levels of excessive ACh were alleviated by BuChE activity^20^. In view of an increased level of BuChE and decreased level of AChE in the progressed AD, development of effective and selective BuChE inhibitor is of vital importance^21^. Besides, BuChE is associated with drug metabolism and detoxification, lipoprotein metabolism and diseases, etc. The crystal structures of two ChEs are very similar, containing a catalytic active site (CAS), a deep gorge and a peripheral anionic site (PAS)^22^. Compared to AChE, a wider space of BuChE in acyl-binding site allows larger substrates to be recognised and hydrolyzed^23^. The structural feature of BuChE provides a reasonable thought to design selective BuChE inhibitors^24–29^. Although there are some types of scaffolds with BuChE inhibition, selective BuChE inhibitors are far from abundance^30^.

**Figure 1.**
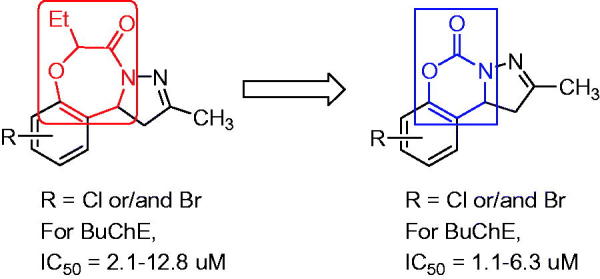
The rational design in this study.

The pyrazole scaffolds are pharmacologically active substances for drug discovery due to their excellent biological and pharmacological activities^31–33^. In our recent works, several pyrazole-containing motifs were designed as potential telomerase inhibitors with anticancer activity^34–36^, selective monoamine oxidase and/or ChE inhibitors for treating Alzheimer’s and Parkinson’s diseases^37^^,^^38^. Furthermore, a series of pyrazole-containing tricyclic scaffolds were found as selective BuChE inhibitors^39^. *h*BuChE (PDB 1P0I)-targeted molecular docking showed that when the seven-membered benzoxazepine ring is replaced by a six-membered benzoxazine ring, the decreased volume may enhance its affinity with target protein (Figure 1). Therefore, we synthesized a series of pyrazolo[1,5-*c*][1,3]benzoxazin-5(5*H*)-one derivatives, and evaluated for their cholinesterase inhibitory activity and the active compounds were used to study the preliminary mechanism.

## 2. Materials and methods

### 2.1. Chemistry

All chemicals, reagents and solvents were purchased from commercial sources and used without further purification. Reactions were checked by thin-layer chromatography (TLC) on precoated silica gel plates (Qingdao Marine Chemical Factory, GF_254_); spots were visualized by UV at 254 nm. Melting points are determined on a XT4MP apparatus (Taike Corp., Beijing, China) and are not corrected. ^1^H NMR and ^13^C NMR spectra were recorded on Bruker AV-600 or AV-300 MHz instruments using DMSO-d_6_ and CDCl_3_ as solvent. Chemical shifts are reported in parts per million (*δ*) downfield from the signal of tetramethylsilane (TMS) as internal standards. Coupling constants are reported in Hz. The multiplicity is defined by s (singlet), d (doublet), t (triplet) or m (multiplet). High resolution mass spectra (HRMS) were obtained on an Agilent 1260-6221 TOF mass spectrometry.

### 2.2. General procedure for the synthesis of compounds (3a–3f)

A series of chalcones (**1**) were synthesized by Claisen–Schmidt condensation of aromatic methyl ketones and salicylaldehyde in alkali ethanol, which were then treated with excess hydrazine hydrate to afford respective pyrazolines (**2**) according to the references^38–40^. Carbonyl diimidazole (CDI, or thiocarbonyl diimidazole, TCDI, 3.0 mmol) was added to CH_2_Cl_2_ (20 ml) solution of compound **2** (2.0 mmol), then the reaction mixture was stirred at room temperature until the disappearance of starting material (monitored by TLC). The reaction mixture was washed with water and brine, dried with anhydrous Na_2_SO_4_, filtrated and concentrated *in vacuo*. The residue was purified by chromatography on a silica gel column (petroleum/EtOAc, 1:1 → 1:2) to give title compounds **3a**–**3f**. Compounds **3a**–**3f** are known ones with no reports about bioactivity^40^.

#### 2.2.1. 2-Phenyl-1,10b-dihydro-5*H*-pyrazolo[1,5-*c*][1,3]benzoxazin-5-one (3a)

White powder, yield: 26%; m.p. 200–202 °C; ^1^H NMR (300 MHz, CDCl_3_) *δ* 7.91–7.84 (m, 2H, Ar[sbond]H), 7.49–7.34 (m, 4H, Ar[sbond]H), 7.22 (m, 3H, Ar[sbond]H), 5.40 (dd, *J* = 12.9, 10.5 Hz, 1H, 10b-H), 3.83 (dd, *J* = 16.3, 10.4 Hz, 1H, 1-Ha), 3.48 (dd, *J* = 16.2, 13.1 Hz, 1H, 1-Hb). ^13^C NMR (75 MHz, CDCl_3_) *δ* 158.2 (C[dbond]O), 150.5 (C[dbond]N), 146.7 (C[sbond]O), 131.3, 130.5, 130.0, 129.0, 127.3, 125.2, 124.8, 122.8, 117.1, 57.6 (C-10b), 38.8 (C-1). TOF-HRMS: *m/z* [M + H]^+^ calcd for C_16_H_13_N_2_O_2_: 265.0972; found: 265.0979.

#### 2.2.2. 2-Phenyl-1,10b-dihydro-5*H*-pyrazolo[1,5-*c*][1,3]benzoxazin-5-thione (3b)

Light yellow powder, yield: 28%; m.p. 189–191 °C; ^1^H NMR (300 MHz, CDCl_3_) *δ* 7.92 (m, 2H, Ar[sbond]H), 7.56–7.38 (m, 4H, Ar[sbond]H), 7.32–7.21 (m, 3H, Ar[sbond]H), 5.37–5.26 (m, 1H, 10b-H), 3.94 (dd, *J* = 16.5, 10.1 Hz, 1H, 1-Ha), 3.55 (dd, *J* = 16.5, 12.4 Hz, 1H, 1-Hb). TOF-HRMS: *m/z* [M + H]^+^ calcd for C_16_H_13_N_2_O_2_: 281.0743; found: 281.0750.

#### 2.2.3. 2-(4-Fluoro)phenyl-1,10b-dihydro-5*H*-pyrazolo[1,5-*c*][1,3]benzoxazin-5-one (3c)

White powder, yield: 21%; m.p. 257–260 °C; ^1^H NMR (300 MHz, CDCl_3_) *δ* 7.92–7.82 (m, 2H, Ar[sbond]H), 7.43–7.22 (m, 3H, Ar[sbond]H), 7.19–7.10 (m, 3H), 5.41 (dd, *J* = 13.0, 10.4 Hz, 1H, 10b-H), 3.81 (dd, *J* = 16.2, 10.4 Hz, 1H, 1-Ha), 3.47 (dd, *J* = 16.2, 13.1 Hz, 1H, 1-Hb). TOF-HRMS: *m/z* [M + H]^+^ calcd for C_16_H_12_FN_2_O_2_: 283.0877; found: 283.0880.

#### 2.2.4. 9-Chloro-2-(4-chloro)phenyl-1,10b-dihydro-5*H*-pyrazolo[1,5-*c*][1,3]benzoxazin-5-one (3d)

White powder, yield: 32%; m.p. 206–208 °C; ^1^H NMR (300 MHz, CDCl_3_) *δ* 7.79 (d, *J* = 8.5 Hz, 2H, Ar[sbond]H), 7.43 (d, *J* = 8.5 Hz, 2H, Ar[sbond]H), 7.35 (dd, *J* = 8.7, 1.8 Hz, 1H, Ar[sbond]H), 7.23 (s, 1H, Ar[sbond]H), 7.14 (d, *J* = 8.7 Hz, 1H, Ar[sbond]H), 5.46–5.33 (m, 1H, 10b-H), 3.80 (dd, *J* = 16.3, 10.5 Hz, 1H, 1-Ha), 3.46 (dd, *J* = 16.3, 13.0 Hz, 1H, 1-Hb). TOF-HRMS: *m/z* [M + H]^+^ calcd for C_16_H_11_Cl_2_N_2_O_2_: 333.0192; found: 333.0196.

#### 2.2.5. 9-Chloro-2-(4-methyl)phenyl-1,10b-dihydro-5*H*-pyrazolo[1,5-*c*][1,3]benzoxazin-5-one (3e)

White powder, yield: 28%; m.p. 186–188 °C; ^1^H NMR (300 MHz, CDCl_3_) *δ* 7.75 (d, *J* = 8.1 Hz, 2H, Ar[sbond]H), 7.34 (dd, *J* = 8.6, 2.1 Hz, 1H, Ar[sbond]H), 7.27 (s, 1H, Ar[sbond]H), 7.24 (d, *J* = 4.4 Hz, 2H, Ar[sbond]H), 7.13 (d, *J* = 8.7 Hz, 1H, Ar[sbond]H), 5.41–5.31 (m, 1H, 10b-H), 3.80 (dd, *J* = 16.2, 10.4 Hz, 1H, 1-Ha), 3.45 (dd, *J* = 16.2, 13.0 Hz, 1H, 1-Hb), 2.41 (s, 3H, CH_3_). ^13^C NMR (100 MHz, CDCl_3_) *δ* 158.3 (C[dbond]O), 150.5 (C[dbond]N), 146.7 (C[sbond]O), 141.9, 129.9, 129.7 (2C), 127.7, 127.3 (2C), 125.2, 124.8, 122.8, 117.1, 57.5 (C-10b), 38.8 (C-1), 21.7 (CH_3_). TOF-HRMS: *m/z* [M + H]^+^ calcd for C_17_H_14_ClN_2_O_2_: 313.0738; found: 313.0743.

#### 2.2.6. 2–(4-Methyl)phenyl-1,10b-dihydro-5*H*-pyrazolo[1,5-*c*][1,3]benzoxazin-5-one (3f)

White powder, yield: 20%; m.p. 186–189 °C; ^1^H NMR (300 MHz, CDCl_3_) *δ* 7.75 (d, *J* = 8.1 Hz, 2H, Ar[sbond]H), 7.41–7.32 (m, 1H, Ar[sbond]H), 7.26–7.21 (m, 4H, Ar[sbond]H), 7.17 (d, *J* = 8.1 Hz, 1H, Ar[sbond]H), 5.36 (dd, *J* = 13.0, 10.4 Hz, 1H, 10b-H), 3.80 (dd, *J* = 16.2, 10.3 Hz, 1H, 1-Ha), 3.43 (dd, *J* = 16.2, 13.1 Hz, 1H, 1-Hb), 2.39 (s, 3H, CH_3_). ^13^C NMR (100 MHz, CDCl_3_) *δ* 158.3 (C[dbond]O), 149.1 (C[dbond]N), 146.2 (C[sbond]O), 142.1, 130.3, 130.0, 129.7 (2C), 127.5, 127.3 (2C), 124.9, 124.4, 118.5, 57.3 (C-10b), 38.7 (C-1), 21.7 (CH_3_). TOF-HRMS: *m/z* [M + H]^+^ calcd for C_17_H_15_N_2_O_2_: 279.1128; found: 279.1134.

### 2.3. General procedure for the synthesis of compounds (6a–6q)

A series of pyrazoline derivatives (**5**) were synthesized by similar synthesis procedure using salicylaldehyde in alkali acetone. CDI (or TCDI, 3.0 mmol) was added to a CH_2_Cl_2_ (20 ml) solution of compound **5** (2.0 mmol), then the reaction mixture was stirred overnight at room temperature. The reaction solution was washed with water and brine, dried with anhydrous Na_2_SO_4_, filtrated and concentrated *in vacuo*. The residue was purified by column chromatography (silica gel, petroleum/EtOAc, 1:1 → 1:2) to give title compounds **6a**–**6q**.

#### 2.3.1. 7,9-Dichloro-2-methyl-1,10b-dihydro-5*H*-pyrazolo[1,5-*c*][1,3]benzoxazin-5-one (6a)

White powder, yield: 27%; m.p. 237–240 °C; ^1^H NMR (300 MHz, CDCl_3_) *δ* 7.42 (d, *J* = 2.2 Hz, 1H, Ar[sbond]H), 7.03 (d, *J* = 1.2 Hz, 1H, Ar[sbond]H), 5.31–5.20 (m, 1H, 10b-H), 3.35 (dd, *J* = 16.8, 10.3 Hz, 1H, 1-Ha), 3.11 (dd, *J* = 16.7, 12.7 Hz, 1H, 1-Hb), 2.20 (s, 3H, CH_3_); ^13^C NMR (75 MHz, CDCl_3_) *δ* 160.3 (C[dbond]O), 145.4 (C[dbond]N), 145.1 (C[sbond]O), 130.3, 130.1, 125.5, 123.34, 123.30, 57.2 (C-10b), 42.4 (C-1), 16.4 (CH_3_). TOF-HRMS: *m/z* [M + H]^+^ calcd for C_11_H_9_Cl_2_N_2_O_2_: 271.0036; found: 271.0038.

#### 2.3.2. 7,9-Dichloro-2-methyl-1,10b-dihydro-5*H*-pyrazolo[1,5-*c*][1,3]benzoxazin-5-thione (6b)

White powder, yield: 35%; m.p. 230–233 °C; ^1^H NMR (300 MHz, CDCl_3_) *δ* 7.43 (d, *J* = 1.7 Hz, 1H, Ar[sbond]H), 7.04 (s, 1H, Ar[sbond]H), 5.25 (t, *J* = 10.9 Hz, 1H, 10b-H), 3.51 (dd, *J* = 17.1, 10.1 Hz, 1H, 1-Ha), 3.22 (dd, *J* = 17.0, 11.9 Hz, 1H, 1-Hb), 2.29 (s, 3H, CH_3_); ^13^C NMR (75 MHz, CDCl_3_) *δ* 174.8 (C[dbond]S), 164.7 (C[dbond]N), 144.6 (C[sbond]O), 130.7, 130.4, 124.8, 123.0, 122.7, 57.4 (C-10b), 42.7 (C-1), 16.7 (CH_3_).TOF-HRMS: *m/z* [M + H]^+^ calcd for C_11_H_9_Cl_2_N_2_OS: 286.9807; found: 286.9806.

#### 2.3.3. 7,9-Dibromo-2-methyl-1,10b-dihydro-5*H*-pyrazolo[1,5-*c*][1,3]benzoxazin-5-one (6c)

White powder, yield: 30%; m.p. 229–232 °C; ^1^H NMR (300 MHz, CDCl_3_) *δ* 7.81–7.67 (m, 1H, Ar[sbond]H), 7.21 (dd, *J* = 2.1, 1.1 Hz, 1H, Ar[sbond]H), 5.26 (dd, *J* = 12.5, 10.4 Hz, 1H, 10b-H), 3.35 (dd, *J* = 16.9, 10.3 Hz, 1H, 1-Ha), 3.12 (dd, *J* = 16.8, 12.6 Hz, 1H, 1-Hb), 2.20 (s, 3H, CH_3_); ^13^C NMR (75 MHz, CDCl_3_) *δ* 160.3 (C[dbond]O), 147.1 (C[dbond]N), 145.1 (C[sbond]O), 135.9, 126.88, 125.8, 117.5, 112.0, 57.1 (C-10b), 42.4 (C-1), 16.4 (CH_3_). TOF-HRMS: *m/z* [M + H]^+^ calcd for C_11_H_9_Br_2_N_2_O_2_: 358.9025; found: 358.9023.

#### 2.3.4. 7,9-Dibromo-2-methyl-1,10b-dihydro-5*H*-pyrazolo[1,5-*c*][1,3]benzoxazin-5-thione (6d)

White powder, yield, 26%; m.p. 268–270 °C; ^1^H NMR (300 MHz, CDCl_3_) *δ* 7.76 (d, *J* = 1.7 Hz, 1H, Ar[sbond]H), 7.21 (d, *J* = 0.8 Hz, 1H, Ar[sbond]H), 5.25–5.15 (m, 1H, 10b-H), 3.47 (dd, *J* = 17.1, 10.0 Hz, 1H, 1-Hb), 3.21 (dd, *J* = 16.2, 11.9 Hz, 1H, 1-Hb), 2.29 (s, 3H, CH_3_).^13^C NMR (75 MHz, CDCl_3_) *δ* 175.1 (C[dbond]S), 164.5 (C[dbond]N), 146.6 (C[sbond]O), 136.3, 126.7, 125.1, 118.2, 111.6, 57.5 (C-10b), 42.9 (C-1), 16.9 (CH_3_). TOF-HRMS: *m/z* [M + H]^+^ calcd for C_11_H_9_Br_2_N_2_OS: 376.8776; found: 376.8777.

#### 2.3.5. 9-Chloro-2-methyl-1,10b-dihydro-5*H*-pyrazolo[1,5-*c*][1,3]benzoxazin-5-one (6e)

White powder, yield: 32%; m.p. 184–186 °C; ^1^H NMR (300 MHz, CDCl_3_) *δ* 7.31 (dd, *J* = 8.7, 2.4 Hz, 1H, Ar[sbond]H), 7.12 (dd, *J* = 2.4, 1.0 Hz, 1H, Ar[sbond]H), 7.09 (d, *J* = 8.8 Hz, 1H, Ar[sbond]H), 5.24 (dd, *J* = 12.6, 10.4 Hz, 1H, 10b-H), 3.34 (dd, *J* = 16.8, 10.3 Hz, 1H, 1-Ha), 3.11 (dd, *J* = 17.9, 12.7 Hz, 1H, 1-Hb), 2.19 (s, 3H, CH_3_); ^13^C NMR (75 MHz, CDCl_3_) *δ* 159.9 (C[dbond]O), 149.1 (C[dbond]N), 146.1 (C[sbond]O), 130.2, 129.9, 124.9, 124.3, 118.5, 57.0 (C-10b), 42.5 (C-1), 16.4 (CH_3_). TOF-HRMS: *m/z* [M + H]^+^ calcd for C_11_H_10_ClN_2_O_2_: 237.0425; found: 237.0430.

#### 2.3.6. 9-Chloro-2-methyl-1,10b-dihydro-5*H*-pyrazolo[1,5-*c*][1,3]benzoxazin-5-thione (6f)

Light yellow powder, yield: 28%; m.p. 220–222 °C; ^1^H NMR (300 MHz, CDCl_3_) *δ* 7.35 (dd, *J* = 8.7, 2.0 Hz, 1H, Ar[sbond]H), 7.18 (d, *J* = 8.7 Hz, 1H, Ar[sbond]H), 7.12 (d, *J* = 1.3 Hz, 1H, Ar[sbond]H), 5.28–5.12 (m, 1H, 10b-H), 3.49 (dd, *J* = 17.1, 10.0 Hz, 1H, 1-Ha), 3.21 (dd, *J* = 17.5, 12.5 Hz, 1H, 1-Hb), 2.29 (s, 3H, CH_3_); ^13^C NMR (75 MHz, CDCl_3_) *δ* 176.1 (C[dbond]S), 164.7 (C[dbond]N), 148.3 (C[sbond]O), 130.9, 130.2, 124.7, 123.5, 118.0, 57.3 (C-10b), 42.9 (C-1), 16.8 (CH_3_). TOF-HRMS: *m/z* [M + H]^+^ calcd for C_11_H_10_ClN_2_OS: 253.0197; found: 253.0201.

#### 2.3.7. 9-Bromo-2-methyl-1,10b-dihydro-5*H*-pyrazolo[1,5-*c*][1,3]benzoxazin-5-one (6g)

White powder, yield: 27%; m.p. 211–213 °C; ^1^H NMR (300 MHz, CDCl_3_) *δ* 7.46 (dd, *J* = 8.7, 1.7 Hz, 1H, Ar[sbond]H), 7.27–7.25 (m, 1H, Ar[sbond]H), 7.04 (d, *J* = 8.7 Hz, 1H, Ar[sbond]H), 5.24 (dd, *J* = 12.6, 10.4 Hz, 1H, 10b-H), 3.33 (dd, *J* = 16.8, 10.3 Hz, 1H, 1-Ha), 3.11 (dd, *J* = 17.9, 12.7 Hz, 1H, 1-Hb), 2.18 (d, *J* = 6.2 Hz, 3H, CH_3_); ^13^C NMR (75 MHz, CDCl_3_) *δ* 159.8 (C[dbond]O), 149.6 (C[dbond]N), 146.0 (C[sbond]O), 132.9, 127.8, 124.8, 118.9, 117.5, 56.9 (C-10b), 42.5 (C-1), 16.4 (CH_3_). TOF-HRMS: *m/z* [M + H]^+^ calcd for C_11_H_10_BrN_2_O_2_: 280.9920; found: 280.9925.

#### 2.3.8. 9-Bromo-2-methyl-1,10b-dihydro-5*H*-pyrazolo[1,5-*c*][1,3]benzoxazin-5-thione (6h)

Light yellow powder, yield: 31%; m.p. 245–247 °C; ^1^H NMR (300 MHz, CDCl_3_) *δ* 7.50 (dd, *J* = 8.7, 1.8 Hz, 1H, Ar[sbond]H), 7.27–7.25 (m, 1H, Ar[sbond]H), 7.14 (d, *J* = 8.7 Hz, 1H, Ar[sbond]H), 5.26–5.11 (m, 1H, 10b-H), 3.47 (dd, *J* = 17.1, 10.0 Hz, 1H, 1-Ha), 3.21 (dd, *J* = 18.1, 12.0 Hz, 1H, 1-Hb), 2.29 (s, 3H, CH_3_); ^13^C NMR (75 MHz, CDCl_3_) *δ* 176.0 (C[dbond]S), 164.2 (C[dbond]N), 148.7 (C[sbond]O), 133.1, 127.5, 123.7, 118.3, 118.1, 57.0 (C-10b), 42.8 (C-1), 16.7 (CH_3_). TOF-HRMS: *m/z* [M + H]^+^ calcd for C_11_H_10_BrN_2_OS: 296.9692; found: 296.9695.

#### 2.3.9. 7-Bromo-9-chloro-2-methyl-1,10b-dihydro-5*H*-pyrazolo[1,5-*c*][1,3]benzoxazin-5-one (6i)

White powder, yield: 29%; m.p. 228–231 °C; ^1^H NMR (300 MHz, CDCl_3_) *δ* 7.59 (d, *J* = 2.2 Hz, 1H, Ar[sbond]H), 7.07 (s, 1H, Ar[sbond]H), 5.31–5.20 (m, 1H, 10b-H), 3.34 (dd, *J* = 16.8, 10.3 Hz, 1H, 1-Ha), 3.11 (dd, *J* = 16.3, 12.8 Hz, 1H, 1-Hb), 2.20 (s, 3H, CH_3_); ^13^C NMR (75 MHz, CDCl_3_) *δ* 160.13 (C[dbond]O), 146.66 (C[dbond]N), 145.15 (C[sbond]O), 133.3, 130.5, 125.3, 123.9, 111.8, 57.2 (C-10b), 42.4 (C-1), 16.4 (CH_3_). TOF-HRMS: *m/z* [M + H]^+^ calcd for C_11_H_9_BrClN_2_O_2_: 314.9530; found: 314.9537.

#### 2.3.10. 7-Bromo-9-chloro-2-methyl-1,10b-dihydro-5*H*-pyrazolo[1,5-*c*][1,3]benzoxazin-5-thione (6j)

White powder, yield: 30%; m.p. 248–250 °C; ^1^H NMR (300 MHz, CDCl_3_) *δ* 7.60 (d, *J* = 2.1 Hz, 1H, Ar[sbond]H), 7.07 (d, *J* = 1.1 Hz, 1H, Ar[sbond]H), 5.23 (t, *J* = 10.9 Hz, 1H, 10b-H), 3.49 (dd, *J* = 17.1, 10.1 Hz, 1H, 1-Ha), 3.21 (dd, *J* = 16.7, 12.2 Hz, 1H, 1-Hb), 2.29 (s, 3H, CH_3_); ^13^C NMR (75 MHz, CDCl_3_) *δ* 175.0 (C[dbond]O), 165.0 (C[dbond]N), 146.0 (C[sbond]O), 133.4, 131.1, 124.7, 123.9, 111.1, 57.6 (C-10b), 42.8 (C-1), 16.9 (CH_3_). TOF-HRMS: *m/z* [M + H]^+^ calcd for C_11_H_9_BrClN_2_OS: 330.9302; found: 330.9306.

#### 2.3.11. 2-Methyl-7-methoxy-1,10b-dihydro-5*H*-pyrazolo[1,5-*c*][1,3]benzoxazin-5-one (6k)

White powder, yield: 27%; m.p. 189–192 °C; ^1^H NMR (300 MHz, CDCl_3_) *δ* 7.12 (t, *J* = 8.0 Hz, 1H, Ar[sbond]H), 6.92 (d, *J* = 8.2 Hz, 1H, Ar[sbond]H), 6.69 (d, *J* = 7.7 Hz, 1H, Ar[sbond]H), 5.25 (dd, *J* = 12.5, 10.5 Hz, 1H, 10b-H), 3.89 (s, 3H, CH_3_), 3.32 (dd, *J* = 16.8, 10.3 Hz, 1H, 1-Ha), 3.10 (dd, *J* = 16.3, 13.3 Hz, 1H, 1-Hb), 2.18 (s, 3H, CH_3_); ^13^C NMR (75 MHz, CDCl_3_) *δ* 159.7 (C[dbond]O), 148.0 (C[dbond]N), 146.3 (C[sbond]O), 139.9, 125.3, 123.9, 115.9, 112.7, 57.3 (C-10b), 56.4 (OCH_3_), 42.7 (C-1), 16.4 (CH_3_). TOF-HRMS: *m/z* [M + H]^+^ calcd for C_12_H_13_N_2_O_3_: 233.0921; found: 233.0925.

#### 2.3.12. 2-Methyl-7-methoxy-1,10b-dihydro-5*H*-pyrazolo[1,5-*c*][1,3]benzoxazin-5-thione (6l)

Light yellow powder, yield: 28%; m.p. 217–219 °C; ^1^H NMR (300 MHz, CDCl_3_) *δ* 7.16 (t, *J* = 8.0 Hz, 1H, Ar[sbond]H), 6.95 (d, *J* = 8.2 Hz, 1H, Ar[sbond]H), 6.68 (d, *J* = 7.7 Hz, 1H, Ar[sbond]H), 5.25–5.10 (m, 1H, 10b-H), 3.91 (s, 3H, CH_3_), 3.47 (dd, *J* = 17.1, 10.0 Hz, 1H, 1-Ha), 3.20 (dd, *J* = 17.1, 12.1 Hz, 1H, 1-Hb), 2.28 (s, 3H, CH_3_); ^13^C NMR (75 MHz, CDCl_3_) *δ* 176.3 (C[dbond]O), 164.5 (C[dbond]N), 147.4 (C[sbond]O), 139.3, 126.1, 123.1, 115.6, 112.9, 57.5 (C-10b), 56.4 (OCH_3_), 43.1 (C-1), 16.8 (CH_3_). TOF-HRMS: *m/z* [M + H]^+^ calcd for C_12_H_13_N_2_O_2_S: 249.0692; found: 249.0694.

#### 2.3.13. 2,9-Dimethyl-1,10b-dihydro-5*H*-pyrazolo[1,5-*c*][1,3]benzoxazin-5-one (6m)

White powder, yield: 31%; m.p. 174–177 °C; ^1^H NMR (300 MHz, CDCl_3_) *δ* 7.12 (d, *J* = 8.3 Hz, 1H, Ar[sbond]H), 7.01 (d, *J* = 8.3 Hz, 1H, Ar[sbond]H), 6.91 (s, 1H, Ar[sbond]H), 5.29–5.15 (m, 1H, 10b-H), 3.32 (dd, *J* = 16.8, 10.2 Hz, 1H, 1-Ha), 3.09 (dd, *J* = 16.3, 13.3 Hz, 1H, 1-Hb), 2.34 (s, 3H, CH_3_), 2.18 (s, 3H, CH_3_); ^13^C NMR (75 MHz, CDCl_3_) *δ* 159.7 (C[dbond]O), 148.3 (C[dbond]N), 146.8 (C[sbond]O), 134.8, 130.3, 125.1, 122.4, 116.7, 57.3 (C-10b), 42.6 (C-1), 20.9 (9-CH_3_), 16.4 (2-CH_3_). TOF-HRMS: *m/z* [M + H]^+^ calcd for C_12_H_13_N_2_O_2_: 217.0972; found: 217.0981.

#### 2.3.14. 2,9-Dimethyl-1,10b-dihydro-5*H*-pyrazolo[1,5-*c*][1,3]benzoxazin-5-thione (6n)

Light yellow powder, yield: 28%; m.p. 188–190 °C; ^1^H NMR (300 MHz, CDCl_3_) *δ* 7.17 (d, *J* = 8.3 Hz, 1H, Ar[sbond]H), 7.12 (d, *J* = 8.3 Hz, 1H, Ar[sbond]H), 6.91 (s, 1H, Ar[sbond]H), 5.20–5.10 (m, 1H, 10b-H), 3.44 (dd, *J* = 17.0, 9.9 Hz, 1H, 1-Ha), 3.20 (dd, *J* = 17.0, 12.2 Hz, 1H, 1-Hb), 2.36 (s, 3H, CH_3_), 2.28 (s, 3H, CH_3_); ^13^C NMR (75 MHz, CDCl_3_) *δ* 177.0 (C[dbond]S), 164.4 (C[dbond]N), 147.8 (C[sbond]O), 135.8, 130.7, 124.9, 121.5, 116.4, 57.6 (C-10b), 43.1 (C-1), 21.1 (9-CH_3_), 16.9 (2-CH_3_). TOF-HRMS: *m/z* [M + H]^+^ calcd for C_12_H_13_N_2_OS: 233.0743; found: 233.0753.

#### 2.3.15. 2-Methyl-1,10b-dihydro-5*H*-pyrazolo[1,5-*c*][1,3]benzoxazin-5-one (6o)

It is a known compound with no reports about bioactivity^41^. Light yellow powder, yield: 32%; m.p. 162–164 °C; ^1^H NMR (600 MHz, CDCl_3_) *δ* 7.37–7.32 (m, 1H, Ar[sbond]H), 7.19 (td, *J* = 7.4, 3.0 Hz, 1H, Ar[sbond]H), 7.16–7.12 (m, 2H, Ar[sbond]H), 5.30–5.22 (m, 1H, 10b-H), 3.35 (dd, *J* = 16.7, 10.2 Hz, 1H, 1-Ha), 3.11 (dd, *J* = 16.1, 12.5 Hz, 1H, 1-Hb), 2.19 (s, 3H, CH_3_); ^13^C NMR (75 MHz, CDCl_3_) *δ* 159.8 (C[dbond]O), 150.5 (C[dbond]N), 146.6 (C[sbond]O), 129.8, 125.1, 124.8, 122.8, 117.0, 57.2 (C-10b), 42.6 (C-1), 16.4 (CH_3_). TOF-HRMS: *m/z* [M + H]^+^ calcd for C_11_H_11_N_2_O_2_: 203.0815; found: 203.0817.

#### 2.3.16. 2-Methyl-1,10b-dihydro-5*H*-pyrazolo[1,5-*c*][1,3]benzoxazin-5-thione (6p)

Light yellow powder, yield: 34%; m.p. 218–220 °C; ^1^H NMR (300 MHz, CDCl_3_) *δ* 7.39 (t, *J* = 7.6 Hz, 1H, Ar[sbond]H), 7.25 (dd, *J* = 7.6, 4.5 Hz, 2H, Ar[sbond]H), 7.13 (d, *J* = 7.8 Hz, 1H, Ar[sbond]H), 5.28–5.14 (m, 1H, 10b-H), 3.49 (dd, *J* = 17.1, 9.9 Hz, 1H, 1-Ha), 3.21 (dd, *J* = 16.9, 12.3 Hz, 1H, 1-Hb), 2.29 (s, 3H, CH_3_). ^13^C NMR (100 MHz, CDCl_3_) *δ* 176.6 (C[dbond]S), 164.7 (C[dbond]N), 149.7 (C[sbond]O), 130.2, 125.8, 124.7, 121.8, 116.5, 57.5 (C-10b), 43.0 (C-1), 16.8 (CH_3_). TOF-HRMS: *m/z* [M + H]^+^ calcd for C_11_H_11_N_2_OS: 219.0587; found: 219.0590.

#### 2.3.17. 7-Bromo-2-methyl-1,10b-dihydro-5*H*-pyrazolo[1,5-*c*][1,3]benzoxazin-5-one (6q)

White powder, yield: 30%; m.p. 218–221 °C; ^1^H NMR (300 MHz, CDCl_3_) *δ* 7.60–7.51 (m, 1H, Ar[sbond]H), 7.06 (dd, *J* = 8.6, 5.3 Hz, 2H, Ar[sbond]H), 5.34–5.21 (m, 1H, 10b-H), 3.37 (dd, *J* = 16.8, 10.3 Hz, 1H, 1-Ha), 3.11 (dd, *J* = 16.7, 12.8 Hz, 1H, 1-Hb), 2.19 (s, 3H, CH_3_); ^13^C NMR (75 MHz, CDCl_3_) *δ* 160.2 (C[dbond]O), 147.7 (C[dbond]N), 145.6 (C[sbond]O), 133.7, 125.9, 124.4, 123.9, 111.0, 57.4 (C-10b), 42.5 (C-1), 16.4 (CH_3_). TOF-HRMS: *m/z* [M + H]^+^ calcd for C_11_H_10_BrN_2_O_2_: 280.9920; found: 280.9918.

### 2.4. AChE/BuChE activity assays

Enzymatic activity assays were performed on AChE from electric eel (C3389-500UN; Sigma) and BuChE from equine serum (C4290-1KU; Sigma), according to Ellman’s method with light modification^42^. The experiment was performed in 48-well plates in a final volume of 500 µl. Each well contained 0.036 U/ml of EeAChE or eqBuChE, and 0.1 M pH = 8 phosphate buffer. They were incubated for 20 min at different concentrations of test compound at 37 °C. Then 0.35 mM acetylthiocholine iodide (ATCh; A5751-1G; Sigma) or 0.5 mM butyrylthiocholine iodide (20820-1G; Sigma) and 0.35 mM 5,5′-ditiobis-2-nitrobenzoico (DTNB; D8130-1G; Sigma) were added. The DTNB produced the yellow anion 5-thio-2-nitrobenzoic acid along with the enzymatic degradation of acetylthiocholine or butyrylthiocholine. The absorbance of each assay was measured at 410 nm after 20 min in a Biotek Synergy HTX Multi-Mode reader. The IC_50_ values were calculated graphically from inhibition curves (log inhibitor concentration vs percent of inhibition). A control experiment was performed under the same conditions without inhibitor and the blank contained buffer, DMSO, DTNB and substrate.

### 2.5. Kinetic studies of eqBuChE inhibition

Kinetic studies were performed with the same test conditions, using six concentrations of substrate (from 0.1 to 1 mM) and four concentrations of inhibitor (0–20 µM). The kinetic type of enzyme inhibition was obtained through the modified Ellman’s method and Lineweaver–Burk secondary plots^43^. Apparent inhibition constants and kinetic parameters were calculated within the “Enzyme kinetics” module of Prism 5.

### 2.6. Cytotoxicity assays

PC12 cells were used to evaluate cell cytotoxicity by methyl thiazolyl tetrazolium (MTT) assay. PC12 cells were cultured in DMEM containing 10% fetal bovine serum, 100 µg/ml streptomycin and 100 units/ml penicillin at 37 °C in a 5% CO_2_ humidified atmosphere, and inoculated at 1 × 10^4^ cells per well in 96-well plate. After cultured for 24 h, the cells were treated with different concentration of compounds in DMEM for 24 h. Then 20 µl of 0.5 mg/ml MTT reagent was added into the cells and incubated for 4 h. After 4 h, cell culture was removed and then 150 µl DMSO was added to dissolve the formazan. The optical density was measured at 570 nm (OD_570_). Cell viability was calculated from three independent experiments. OD_570_ of formazan in blank group was set as 100% of viability. Cell viability (%) = compound (OD_570_)**/**blank (OD_570_) × 100%.

Blank: cultured with fresh medium only.

Compound: treated with compounds or donepezil.

### 2.7. Neuroprotective effect

The differentiated PC12 cells and oxidative agent H_2_O_2_ were used as *in vitro* model to assess neuronal differentiation and neurobiochemical and neurobiological properties^39^^,^^44^. Differentiated PC12 cells were incubated with different concentrations of compounds **6a** and **6g** for 3 h before treatment with H_2_O_2_ (300 µM). Cell viability was measured after 24 h using MTT method. Briefly, 20 µL of 0.5 mg/ml MTT reagent was added into the cells and incubated for 4 h. After 4 h, cell culture was removed and then 150 µl DMSO was added to dissolve the formazan. OD_570_ value was measured at 570 nm on the Biotek Synergy HTX Multi-Mode reader. Results were adjusted by OD_570_ value in the blank.

### 2.8. Log *P* assessment

Log *P*, defined as the logarithm of octanol–water partition coefficient, is an important parameter to evaluate lipophilicity of compounds^45^. It can be calculated by determining the concentration of compound in octanol phase and water phase until the partition equilibrium was completed. In this work, log *P* of title compounds was measured by the shake flask method with slight modification. PBS (pH = 7.4) was used as the water. Both the octanol and the aqueous phase were saturated with each other before use. The assay mixture containing test compounds was shaken at 37 °C. After 24 h, the mixture was centrifuged at 4,000 rpm for 30 min, followed by the measurement with UV spectrophotometer. Experiments were conducted in triplicate and log *p* values were calculated.

### 2.9. Molecular docking study

In order to further understand SAR, based on the X-ray crystal structure of human BuChE (*h*BuChE PDB ID: 1P0I), molecular docking was performed on the binding model using the Discovery Studio 2017R2 software^22^^,^^23^. A structure based *in silico* procedure was applied to discover the binding modes of the active compounds at BuChE enzyme active site. The CDOCKER of Discovery Studio 2017R2 (DS) was conducted to explain SAR of series compounds and further guide the design of more effective and specific BuChE inhibitors. The ligand binding to the crystal structure of *h*BuChE (PDB ID: 1P0I) was selected as template. The target enzyme was prepared with Prepare Protein of DS to ensure the integrity of target. The ligand was processed by Full Minimization of the Small Molecular in DS. Then title compounds were docked into the active site of protein using CDOCKER. The view results of docking were extracted after the program running end, each docking result was analyzed for interaction and their different pose. The binding energies of most potent compounds were clearly observed and tabulated in Table 2. The lowest -CDOCKER_INTERACTION_ENERGY values of those poses were regarded as the most stable and picked to analyze binding interactions with target enzyme visualized.

## 3. Results and discussion

### 3.1. Chemistry

**Scheme 1.**
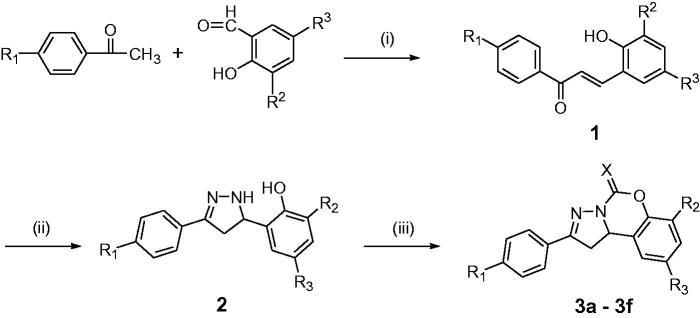
Synthesis of compounds **3a–3f**. Reagents and conditions: (i) 40% NaOH solution, EtOH, 60 °C; (ii) N_2_H_4_·H_2_O, EtOH, reflux; (iii) *N*,*N*′-carbonyldiimidazole or 1,1′-thiocarbonyldiimidazole, CH_2_Cl_2_, r.t.

**Scheme 2.**
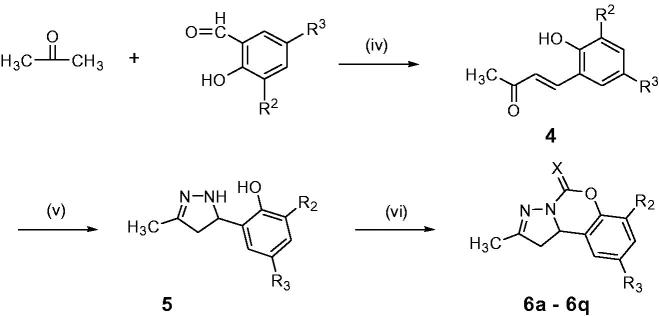
Synthesis of compounds **6a–6q**. Reagents and conditions: (iv) 40% NaOH solution, 60 °C; (v) N_2_H_4_·H_2_O, EtOH, reflux; (vi) *N*,*N*′-carbonyldiimidazole or 1,1′-thiocarbonyldiimidazole, CH_2_Cl_2_, r.t.

According to recent works^37–39^, pyrazolo[1,5-*c*][1,3]benzoxazepin-5(5*H*)-one scaffolds (**3a**–**3f** and **6a**–**6q**, as shown in Table 1) were synthesized by the protocol outlined in Schemes 1 and 2. The intermediate 2-pyrazolines (**2** and **5**) were synthesized through the cyclization reaction of excess hydrazine hydrate with the styrene ketones, which were obtained from Claisen–Schmidt condensation. The title compounds **3** and **6** were respectively synthesized by the intramolecular coupled reaction of compounds **2** and **5** using carbonyl diimidazole (or thiocarbonyl diimidazole) as the couplant in CH_2_Cl_2_. The total yield was 20%–35%.

**Table 1.** Chemical structures of compounds **3a**–**3f** and **6a**–**6q** and their inhibitory activities against EeAChE and eqBuChE^a^


Compound	X	R_1_	R_2_	R_3_	IC_50_, µM (or inhibition % at 20 µM)	
					AChE^b^	BuChE^c^
**3a**	O	Ph	H	H	^d^	9.47 ± 0.23
**3b**	S	Ph	H	H	^d^	9 ± 3%
**3c**	O	4-F[sbond]Ph	H	H	15 ± 3%	15 ± 2%
**3d**	O	4-Cl[sbond]Ph	H	Cl	39 ± 1%	41 ± 6%
**3e**	O	4-CH_3_[sbond]Ph	H	Cl	32 ± 2%	32 ± 5%
**3f**	O	4-CH_3_[sbond]Ph	H	H	48 ± 3%	32 ± 2%
**6a**	O	CH_3_	Cl	Cl	39 ± 3%	1.06 ± 0.11
**6b**	S	CH_3_	Cl	Cl	25 ± 1%	^d^
**6c**	O	CH_3_	Br	Br	10.71 ± 0.64	1.63 ± 0.42
**6d**	S	CH_3_	Br	Br	26 ± 3%	^d^
**6e**	O	CH_3_	H	Cl	16 ± 1%	6.29 ± 0.65
**6f**	S	CH_3_	H	Cl	18 ± 2%	^d^
**6g**	O	CH_3_	H	Br	26 ± 1%	1.63 ± 0.34
**6h**	S	CH_3_	H	Br	27 ± 2%	^d^
**6i**	O	CH_3_	Br	Cl	13.03 ± 0.32	2.44 ± 0.44
**6j**	S	CH_3_	Br	Cl	14 ± 2%	^d^
**6k**	O	CH_3_	OCH_3_	H	35 ± 3%	14 ± 3%
**6l**	S	CH_3_	OCH_3_	H	30 ± 2%	2 ± 2%
**6m**	O	CH_3_	H	CH_3_	^d^	14 ± 1%
**6n**	S	CH_3_	H	CH_3_	15 ± 2%	^d^
**6o**	O	CH_3_	H	H	^d^	50.08 ± 4.3%
**6p**	S	CH_3_	H	H	^d^	^d^
**6q**	O	CH_3_	Br	H	^d^	3.22 ± 1.06
**donepezil**					0.014 ± 0.001	10.38 ± 0.20

^a^Each IC_50_ value is the mean ± SEM from three experiments (*n* = 3).

^b^AChE from electric eel.

^c^BuChE from horse serum.

^^d^^No inhibitory activity (%) against either EeAChE or eqBuChE at 20 µM.

### 3.2. Biological activity

The inhibitory activities of title compounds against AChE and BuChE were assessed by modified Ellman’s method. The IC_50_ values were obtained and compared to the reference donepezil, a selective AChE inhibitor, which was the only one of four FDA-approved AChEIs. The IC_50_ values of all synthesized compounds against EeAChE and eqBuChE are summarized in Table 1.

According to the biological activity results, 23 tricyclic compounds showed inhibitory activities against cholinesterases. Compared to the reference donepezil, some compounds showed potent inhibitory activity against BuChE, but only moderate against AChE. From Table 1, it was obvious from the data that compounds **6a**, **6c** and **6g** exhibited the best activities against BuChE with IC_50_ values of 1.06, 1.63 and 1.63 µM, respectively, surpassing that of control donepezil (IC_50_ = 10.38 µM). On inspection of the chemical structures, it can be concluded that BuChE inhibitory activity was related to the substituent groups at C2, C5, C7 and C9 positions of benzoxazinone moiety. When carbonyl at C5 position was substituted by sulfur carbonyl, BuChE inhibitory activity significantly decreased. Further, for compounds with carbonyl at C5 position, halogen substituents at the benzene ring have great influence on the BuChE inhibition, for example, compounds **6a**, **6c**, **6e**, **6g**, **6i** and **6q** with 7- or/and 9-halogen substitutions at the benzene ring exhibited better in terms of potency than the corresponding compounds **6k**, **6m** and **6o** with methyl or methoxyl substituents, except for compound **3a**. Compound **6a** with 7,9-dichloro substituents showed the best BuChE inhibitory activity (IC_50_ = 1.06 µM) than the non-halogen substituted compounds **6k** and **6m** (inhibition rate at 20 µM < 20%). The SAR was observed in our recent work^39^. The dehydroevodiamine-derived tri- and tetracyclic compounds also showed potent inhibitory activity and selectivity towards BuChE^30^. The polycyclic scaffold could be used to design selective BuChE inhibitors.

### 3.3. Kinetic study of eqBuChE inhibition

**Figure 2.**
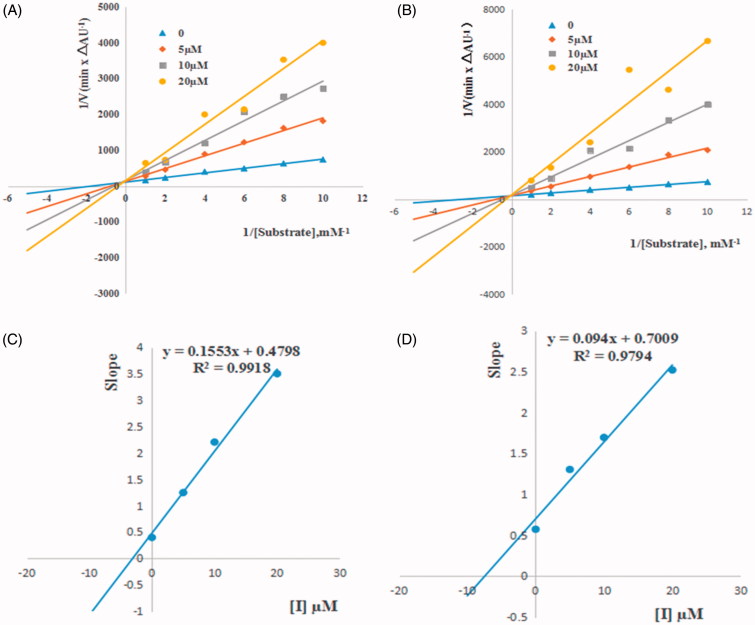
Lineweaver − Burk plots of esBChE inhibition kinetics of compounds **6a** (A), **6g** (B). The Lineweaver–Burk secondary plots of compounds **6a** (C) and **6g** (D). Reciprocals of enzyme activity (esBuChE) vs. reciprocals of substrate (butyrylthiocholine iodide) concentration in the presence of different concentrations (0−20 µM) of inhibitor. Inset: Concentrations used for inhibitor are coded with different graphic symbols.

The kinetic studies were carried out at three fixed inhibitor concentrations (5, 10 and 20 µM). As shown in Figure 2(A,B), for compounds **6a** and **6g**, overlaid reciprocal Lineweaver–Burk plots showed that both slopes (decreased *V*_max_) and intercepts (higher *K*_m_) increase with the increase of inhibitor concentration, which trend is usually ascribed to a mixed-type inhibition. The dissociation constants *K*_i_ for compounds **6a** (Figure 2(C)) and **6d** (Figure 2(D)) were estimated to be 7.46 and 3.09 µM, respectively.

### 3.4. Cytotoxicity assays and neuroprotective effect

**Figure 3.**
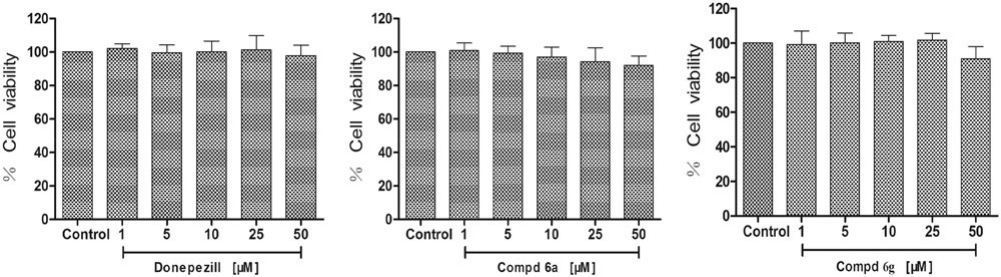
Cytotoxicity of compounds **6a**, **6g** and donepezil tested at concentrations in the range 1–50 µM in PC12 cell lines for 24 h. Untreated cells were used as control. Results are expressed as percentage of cell survival vs. untreated cell (control) and shown as mean ± SD (*n* = 3).

**Figure 4.**
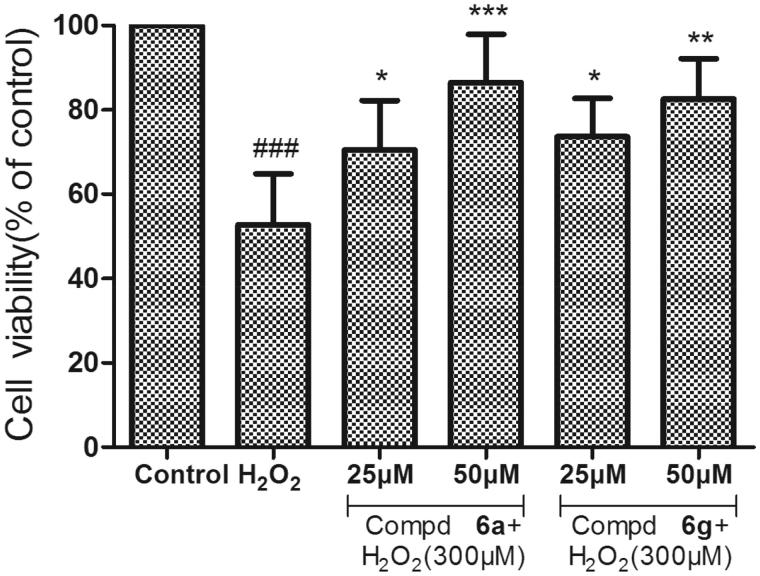
Neuroprotective effect on PC12 cell lines of compounds **6a** and **6g**. After 24 h incubation at different concentration (25 and 50 µM) with H_2_O_2_ (300 µM). Untreated cells were used as control. Results represent mean ± SD (*n* = 3). Statistical significance was calculated using one-way ANOVA and Bonferroni *post hoc* tests. ###*p* < .001 compared with the control group; *p < .05 compared with H_2_O_2_ group.

The neuroprotective activity of compounds **6a**, **6g** and donepezil against oxidative stress-induced cell death in differentiated PC12 neurons was assayed. The differentiated PC12 cells were pretreated with three concentrations (10, 25 and 50 µM) for 3 h, before treatment with H_2_O_2_ (300 µM), and cell viability was measured after 24 h using MTT method. As shown in Figure 3, Compounds **6a**, **6g** and donepezil at the test concentrations (1–50 µM) had no obvious cytotoxicity in PC-12 cells, and the relative cell viabilities of treated cells were all more than 90%. Neuroprotective activity of compounds **6a** and **6g** against eqBuChE was evaluated by subjecting PC12 cells to H_2_O_2_-induced damage. As shown in Figure 4, compared to control group, the percent of cell viability was calculated. Compounds **6a** and **6g** exhibited remarkable neuroprotective activity at 25 µM (cell viability >70%, and *p* < .05 vs. H_2_O_2_ treatment alone).

### 3.5. Log *P* assessment

As a potential compound for treatment of AD, log *P* (octanol–water partition coefficient) was thought as an important physical and chemical parameter to predict the ability to cross blood brain barrier (BBB). The log *P* with optimum central nervous system (CNS) penetration was around 2.0 ± 0.7. The log *P* values of active compounds **6a**, **6c** and **6g** were 1.50, 1.48 and 1.63, respectively, which indicated that the active compounds had sufficient lipophilicity to pass the BBB *in vivo*.

### 3.6. Molecular docking

The results of docking calculation in Table 2 showed that compounds **6a–6q** had good binding affinity to BuChE and their CDOCKER_INTERACTION_ENERGY had almost the same trend as the BuChE inhibitory activities, which further proved the correlation between BuChE inhibitory activity and binding energy. Especially, when carbonyl at C5 position was substituted by sulfur carbonyl, the binding energy significantly decreased.

**Table 2.** -CDOCKER_INTERACTION_ENERGY of title compounds **6a**–**6q** and 1P0I

Compound	-CDOCKER_INTERACTION_ENERGY Δ*G* (kcal/mol)
**6a**	34.3548
**6b**	28.6536
**6c**	33.1657
**6e**	30.9456
**6g**	32.8973
**6i**	32.2189
**6o**	27.7660
**6q**	31.5867

**Figure 5.**
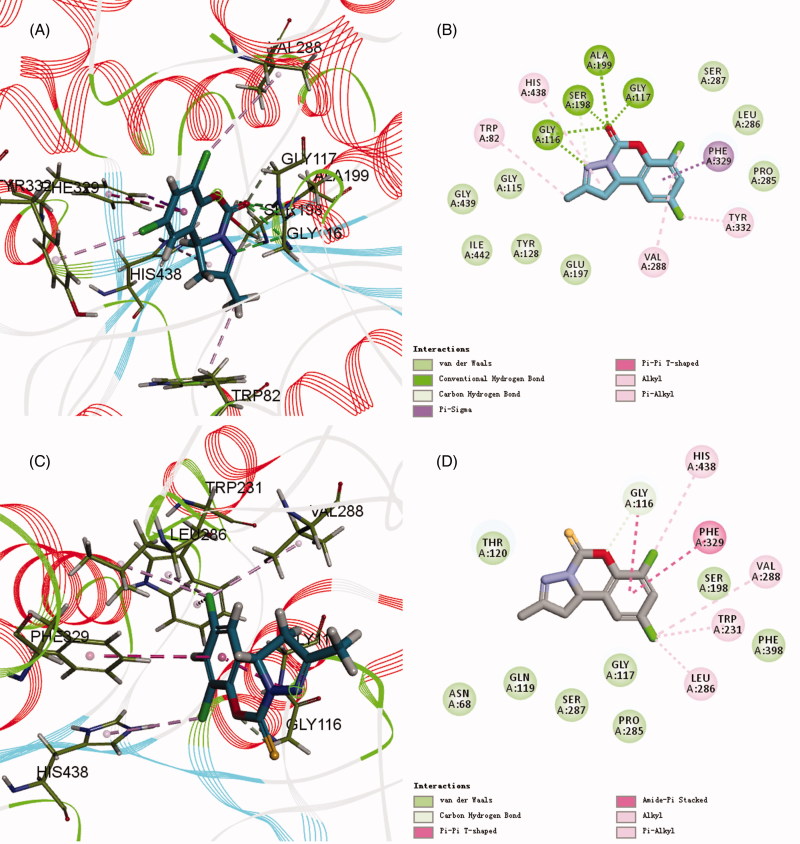
(A,C) 3D mode of interaction of compounds **6a** and **6b** and BuChE (PDB code: 1P0I) analysed by Discovery Studio 2017R2. Conventional hydrogen bond and carbon hydrogen bond and alkyl as well as Pi–alkyl are shown by green, light green and pink, respectively. (B,D) Two dimensional mode of interaction of compounds **6a** and **6b** and BuChE (PDB code: 1P0I) analysed by Discovery Studio 2017R2.

**Figure 6.**
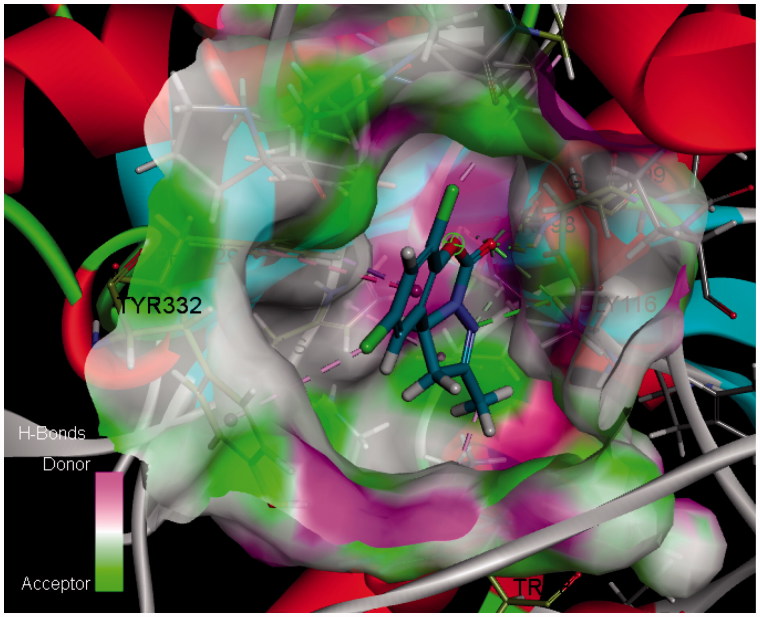
The H-Bonds surface of compound **6a** and BuChE (PDB code: 1P0I) analysed by Discovery Studio 2017R2.

Among them, compound **6a** had the maximum -CDOCKER_INTERACTION_ENERGY and highest activity, while compound **6b** with lower energy exhibited lower activity. The 2D and 3D binding models of compound **6a** and **6b** with BuChE are depicted in Figure 5. Compound **6a** (Figure 5(A,B)) was well accommodated into BuChE via five conventional hydrogen bonds between the C[dbond]O group and Gly116 (distance = 2.67 Å), Gly117 (distance = 1.98 Å), Ala199 (distance = 2.49 Å), Ser198 (distance = 2.17 Å), and between the N atom and Gly116 (distance = 2.67 Å); three carbon hydrogen bond interactions between the C[dbond]O group and CH_2_ group of Ser198 and Gly117, and between the N atom and the CH_2_ group of Ser198; one Pi–Sigma interaction between benzene ring and the aromatic ring of Phe329; one Alkyl interaction between 7-Cl and the end group of Val288; three Pi–Alkyl interactions between 2-CH_3_ and the end group of Trp82, His438 and between 9-Cl and the end group of Tyr332. However, compound **6b** (Figure 5(C,D)) could also be accommodated into BuChE via one hydrogen bond between the O atom and Gly116 (distance = 2.67 Å); one Amide–Pi Stacked interaction between benzene ring and the amide of Gly116; one Pi–Pi shaped interaction between benzene ring and the aromatic ring of Phe329; two Alkyl interactions between 9-Cl and the end group of Val288, Leu286; three Pi–Alkyl interactions between 9-Cl and the end group of Trp231, and between 7-Cl and the end group of His438. Furthermore, the H-bonds surface of compound **6a** and BuChE is also depicted in Figure 6. The above results, along with the biological assay data, suggested that compound **6a** possessed the best inhibitory activity, which will help us carry out the next structure optimization.

## 4. Conclusions

Based on the structural analysis of BuChE-targeted tricyclic scaffolds, we reported design and synthesis of a series of tricyclic pyrazolo[1,5-*c*][1,3]benzoxazin-5(5*H*)-one derivatives and evaluated *in vitro* AChE and BuChE activities. Compounds with 5-carbonyl and 7- or/and 9-halogen substitutions showed potent BuChE inhibitory activity, among them, compounds **6a**, **6c** and **6g** showed the best BuChE inhibition (IC_50_ = 1.06, 1.63 and 1.63 µM, respectively). The SARs analysis showed that (i) BuChE inhibitory activity significantly decreased for compounds with 5-sulfurcarbonyl; (ii) compounds bearing 5-carbonyl and halogen substituents at the benzene ring had better BuChE inhibitory activity; (iii) the volumes of the substituted groups at the C2 position have influences for the BuChE activity. Kinetic studies revealed that compounds **6a** and **6g** showed a mixed-type inhibition against BuChE (*K*_i_ = 7.46 and 3.09 µM, respectively). The active compounds were found to be nontoxic at their effective concentrations and to have sufficient lipophilicity to pass the BBB *in vivo*. Compounds **6a** and **6g** had remarkable neuroprotective activity. Docking results showed that the synthesized compounds had same binding orientation within the active site of target enzyme. Compound **6a** could be accommodated into BuChE via five hydrogen bonds with Gly116, Gly117, Ala199, Ser198 and Gly116, one Pi–Sigma interaction and three Pi–Alkyl interactions. The active compounds may be developed as selective BuChE inhibitors against progressive neurodegenerative disorder.

## Supplementary Material

Supplemental Material
