# Supplementary material for: Design, synthesis and biological evaluation of tricyclic pyrazolo[1,5-c][1,3]benzoxazin-5(5H)-one scaffolds as selective BuChE inhibitors
Source: J Enzyme Inhib Med Chem. 2018 Oct 4;33(1):1506–15. doi: 10.1080/14756366.2018.1488696 (PMC6179045; doi:10.1080/14756366.2018.1488696)
Supplement: Supplemental Material [file IENZ_A_1488696_SM2905.pdf]

# Design, synthesis and biological evaluation of tricyclic pyrazolo[1,5-*c*][1,3]benzoxazin-5(5H)-one scaffolds as selective BuChE inhibitors

Guo-liang Qiu[a,*], Shao-sheng He[a,c,*], Shi-chao Chen[a], Bo Li[a], Hui-hui Wu[b], Jing Zhang[b] & Wen-jian Tang[a]

[a]School of Pharmacy, Anhui Medical University, Hefei 230032, PR China; [b]Anhui Prevention and Treatment Center for Occupational Disease, Anhui No. 2 Province People's Hospital, Hefei 230022, PR China; [c]Lujiang County People's Hospital, Anhui, Lujiang 231500, China

## Content

**Copies of NMR spectra of compounds 3a–3f and 6a–6q** **S2–S26**

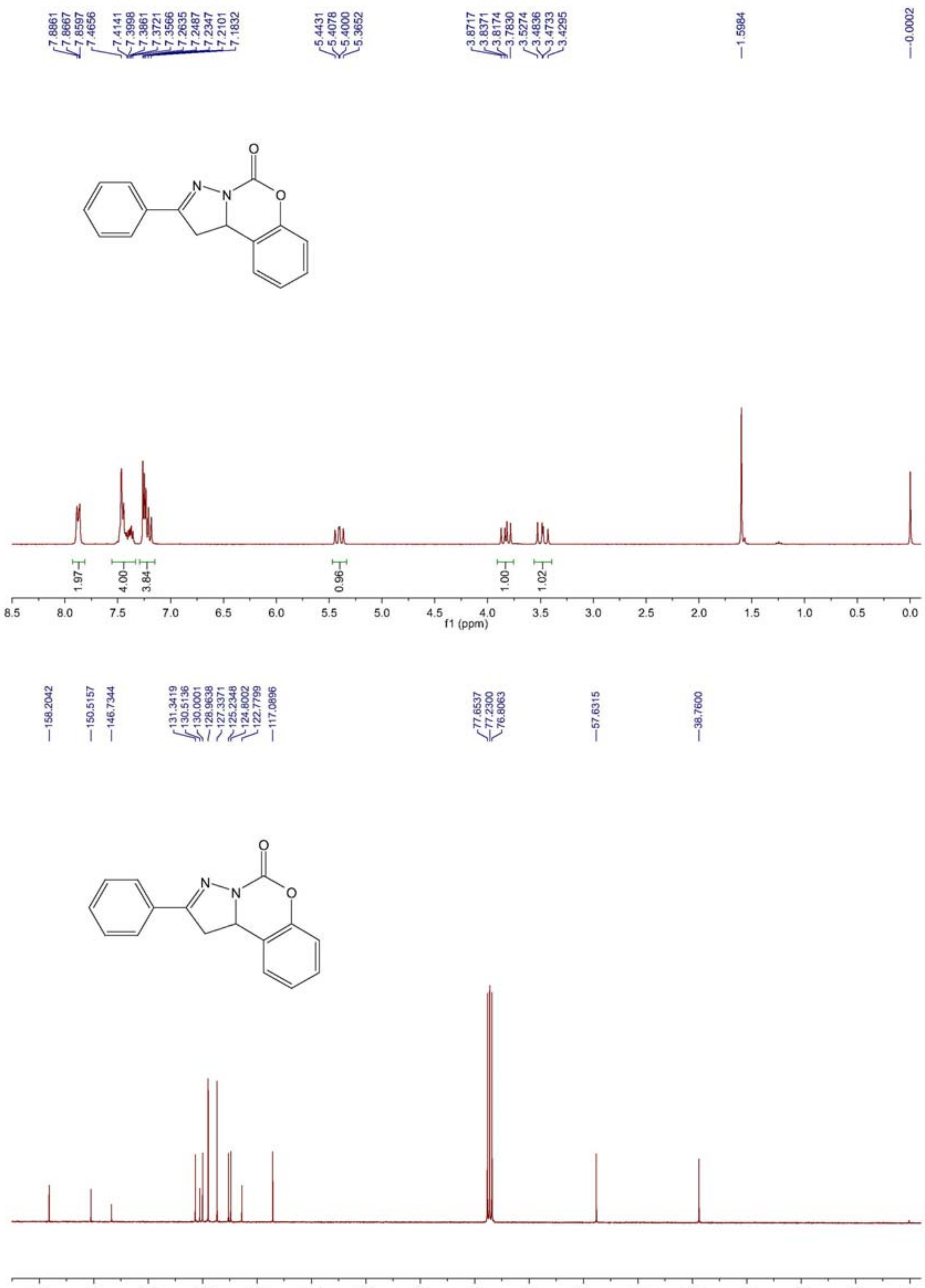

**Figure S1.** $^{1}H$-NMR and $^{13}C$-NMR spectra of compound **3a**.

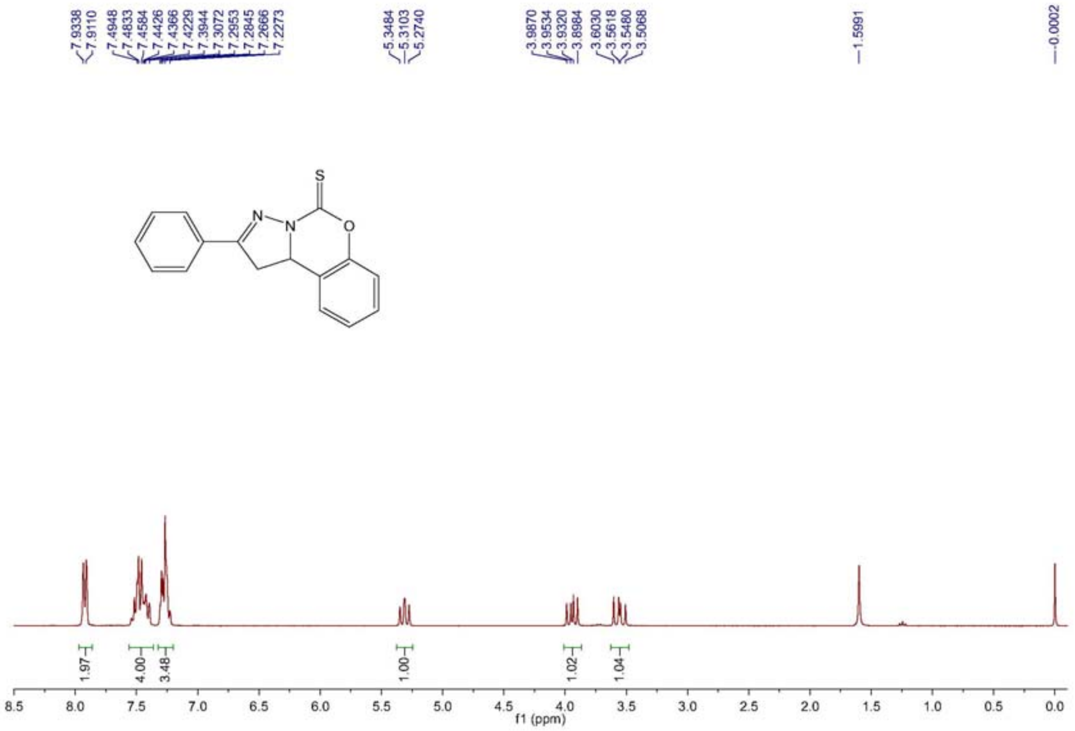


**Figure S2.** $^{1}$H-NMR spectrum of compound **3b**.

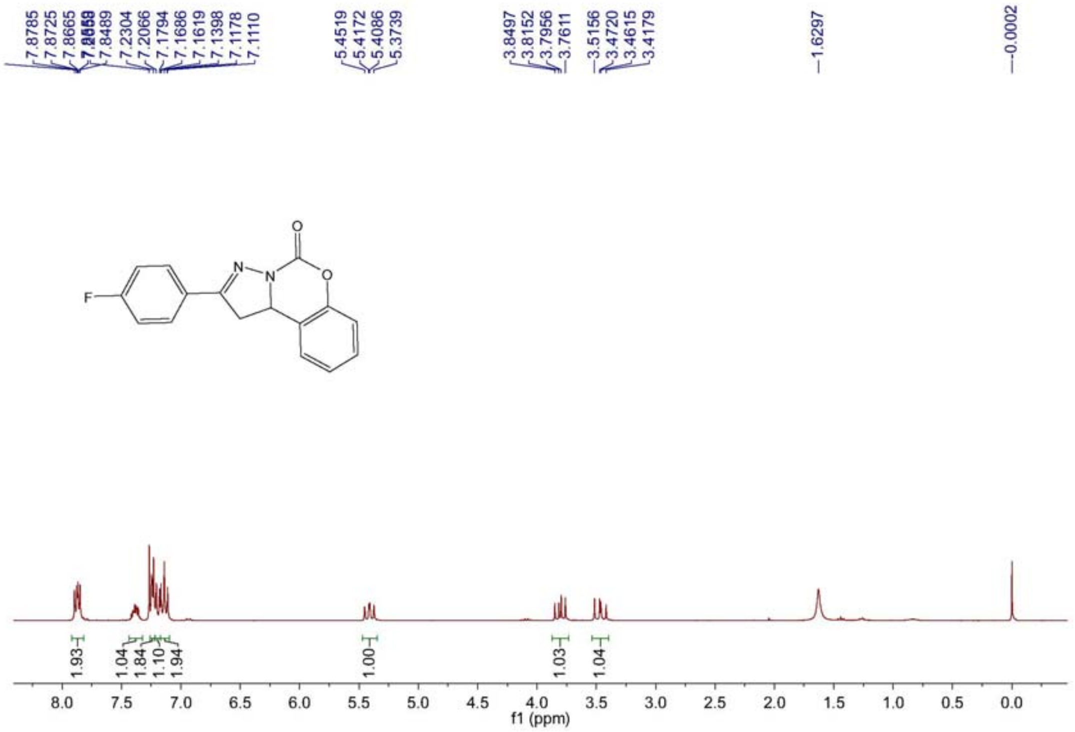


**Figure S3.** $^{1}$H-NMR spectrum of compound **3c**.

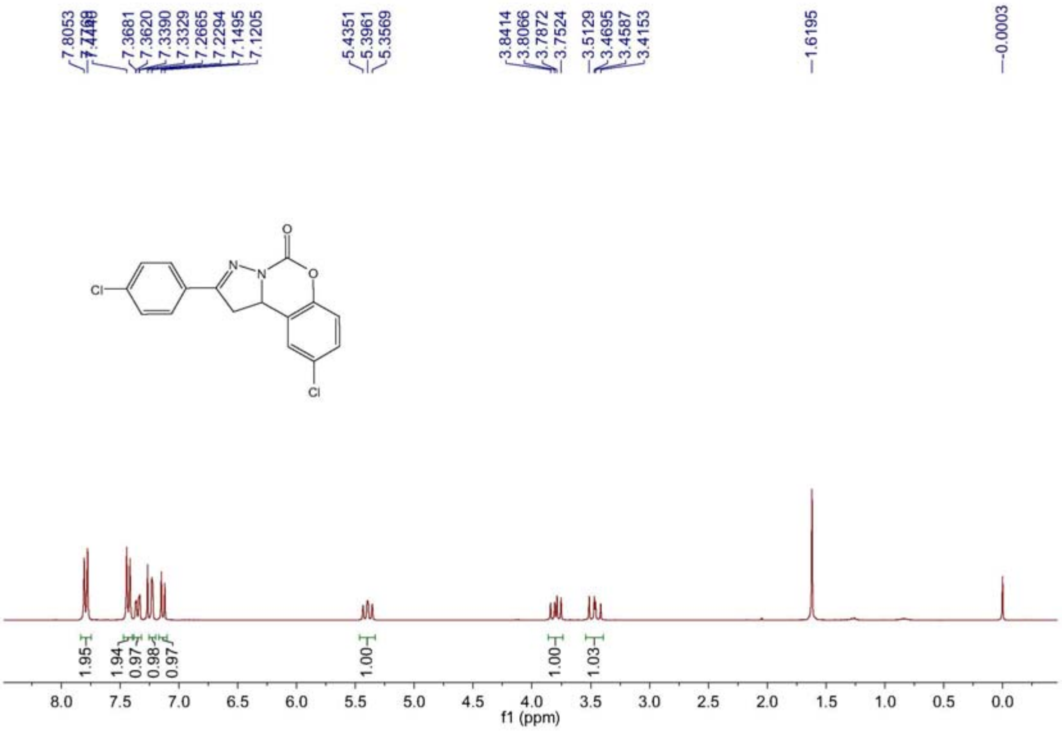

**Figure S4.** $^1$H-NMR spectrum of compound **3d**.

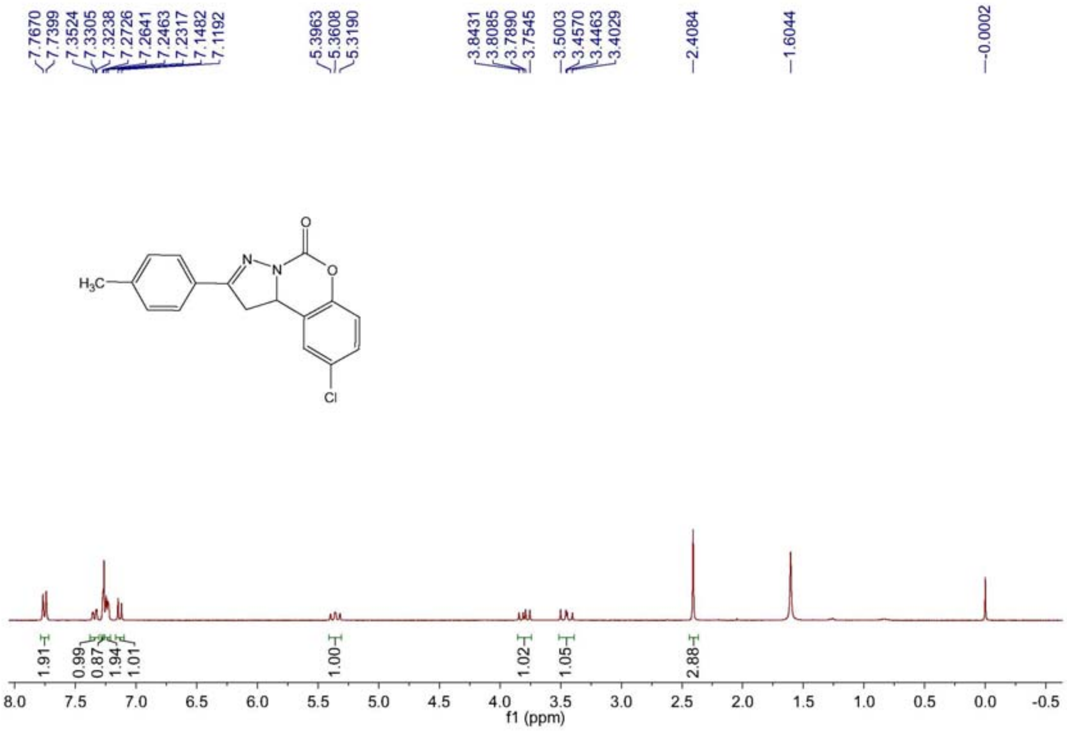


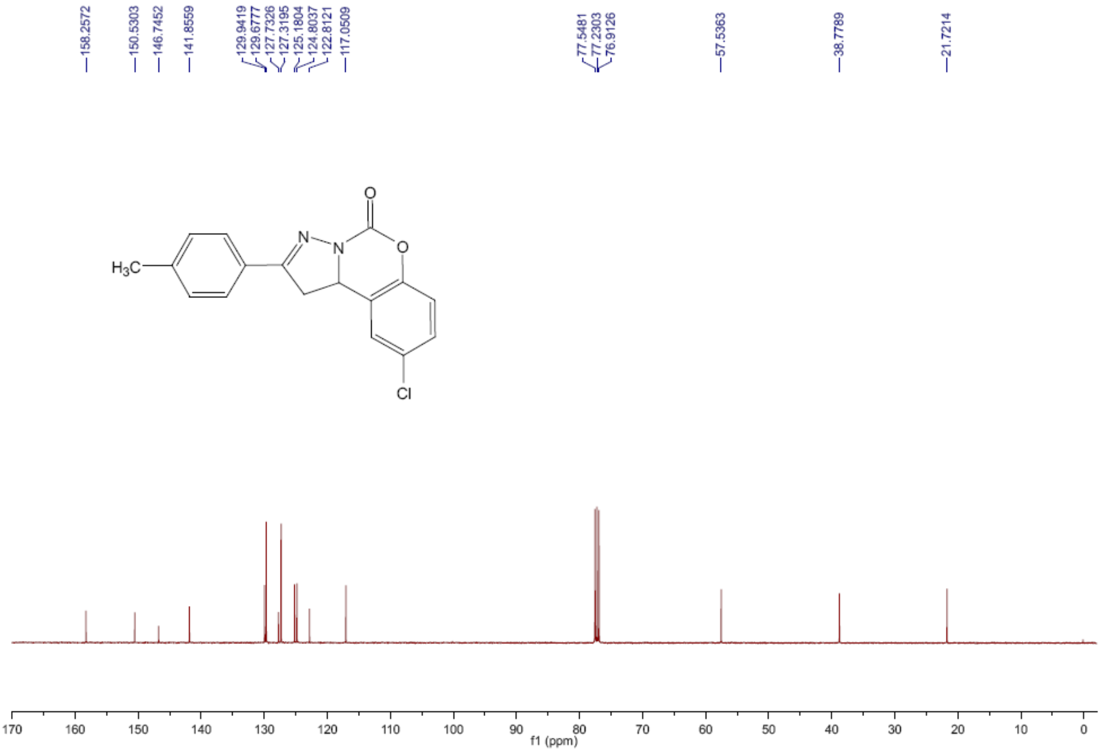


**Figure S5.** $^1$H-NMR spectrum of compound **3e**.

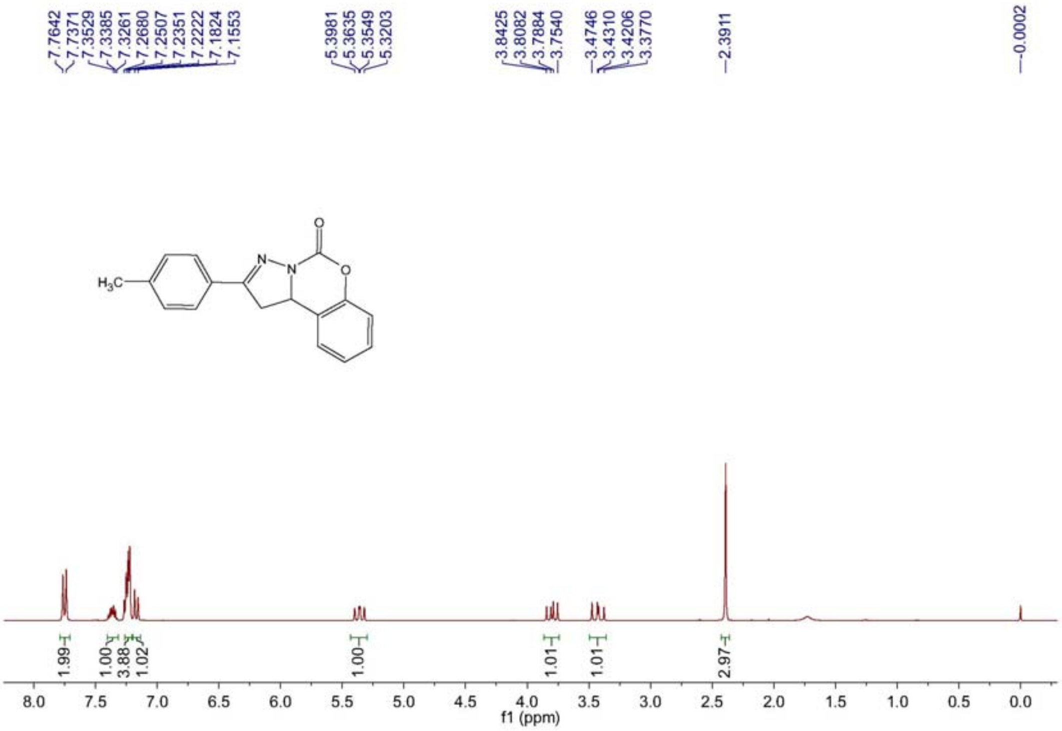
7.7642
7.7371
7.3529
7.3385
7.3261
7.2680
7.2507
7.2351
7.2222
7.1824
7.1553
5.3981
5.3635
5.3549
5.3203
3.8425
3.8082
3.7884
3.7540
3.4746
3.4310
3.4206
3.3770
2.3911
0.0002
H3C
N
N
O
O
1.99
1.00
3.88
1.02
1.00
1.01
1.01
2.97
f1 (ppm)

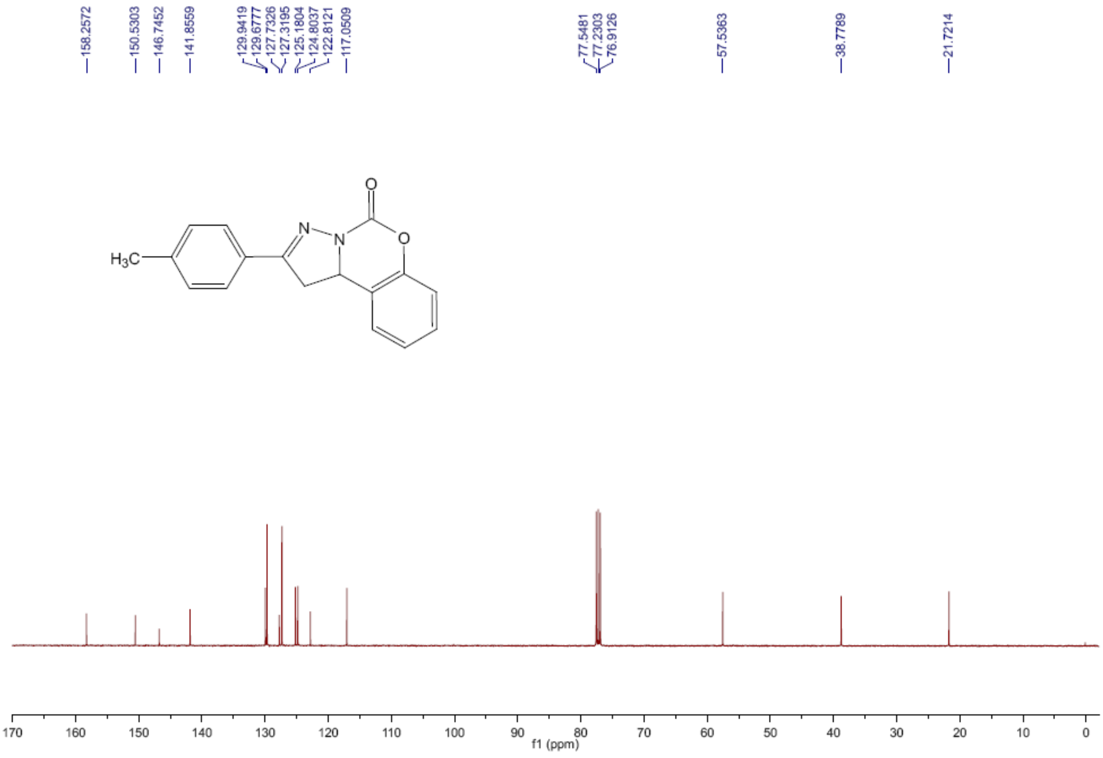
158.2572
150.5303
146.7452
141.8559
129.9419
129.6777
127.7326
127.3195
125.1804
124.8037
122.8121
117.0509
77.5481
77.2303
76.9126
57.5363
38.7789
21.7214
H3C
N
N
O
O
f1 (ppm)

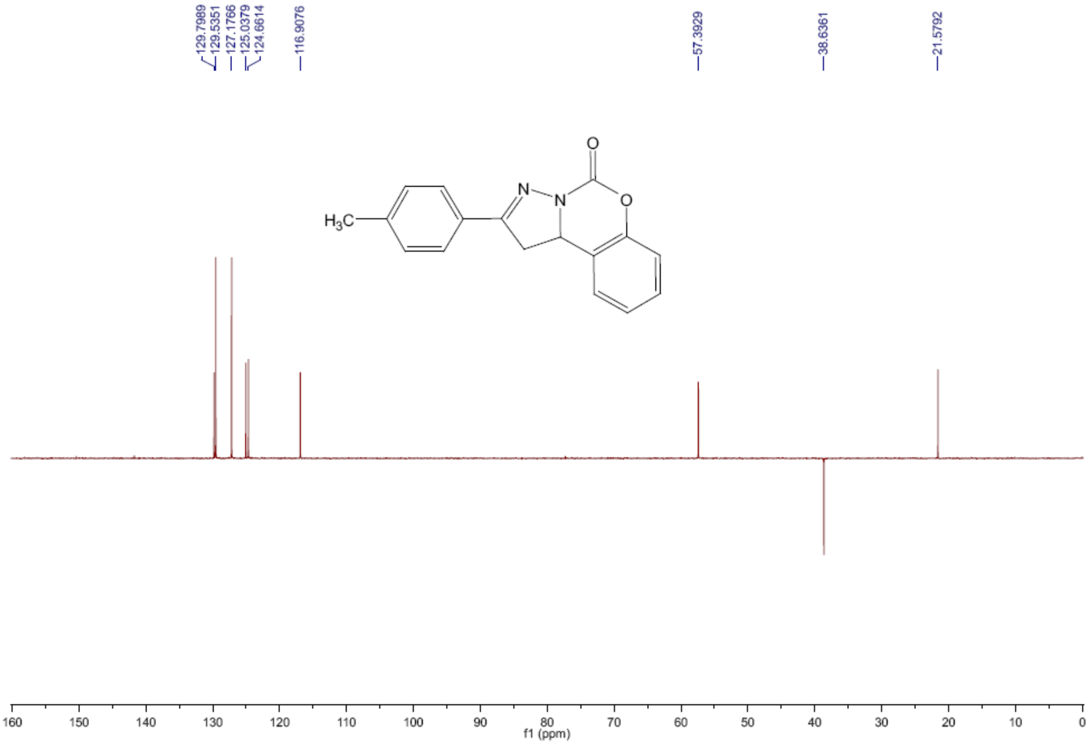


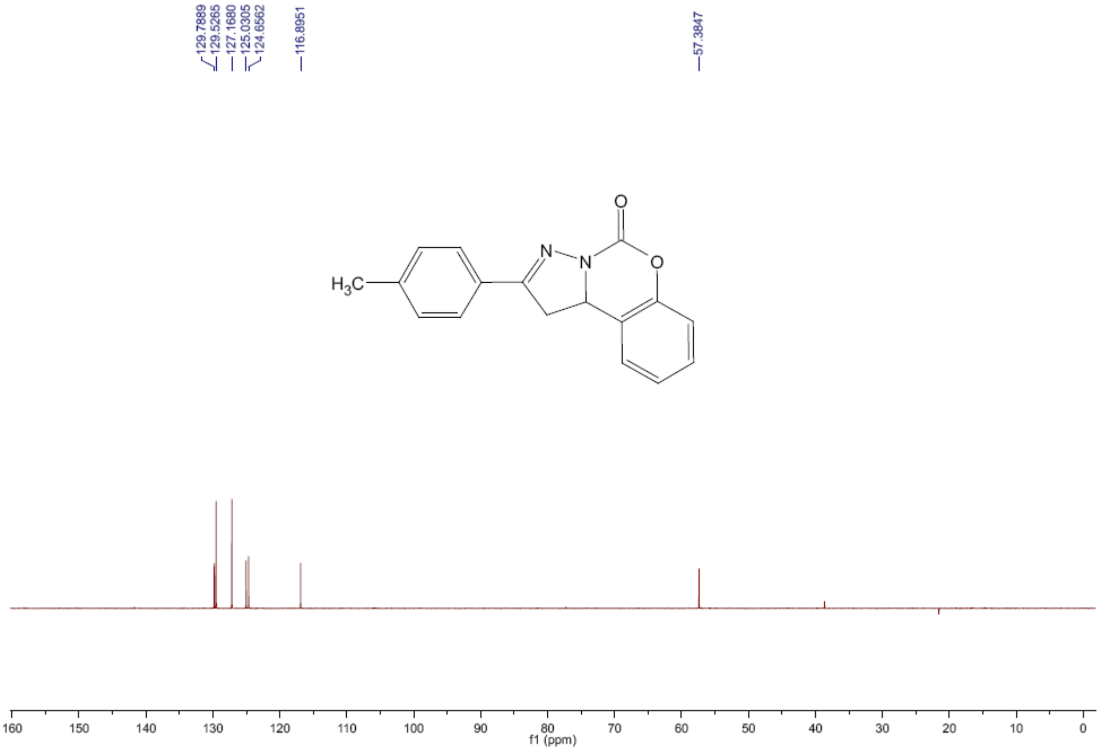


**Figure S6.** $^{1}$H-NMR, $^{13}$C-NMR and DEPT spectra of compound **3f**.

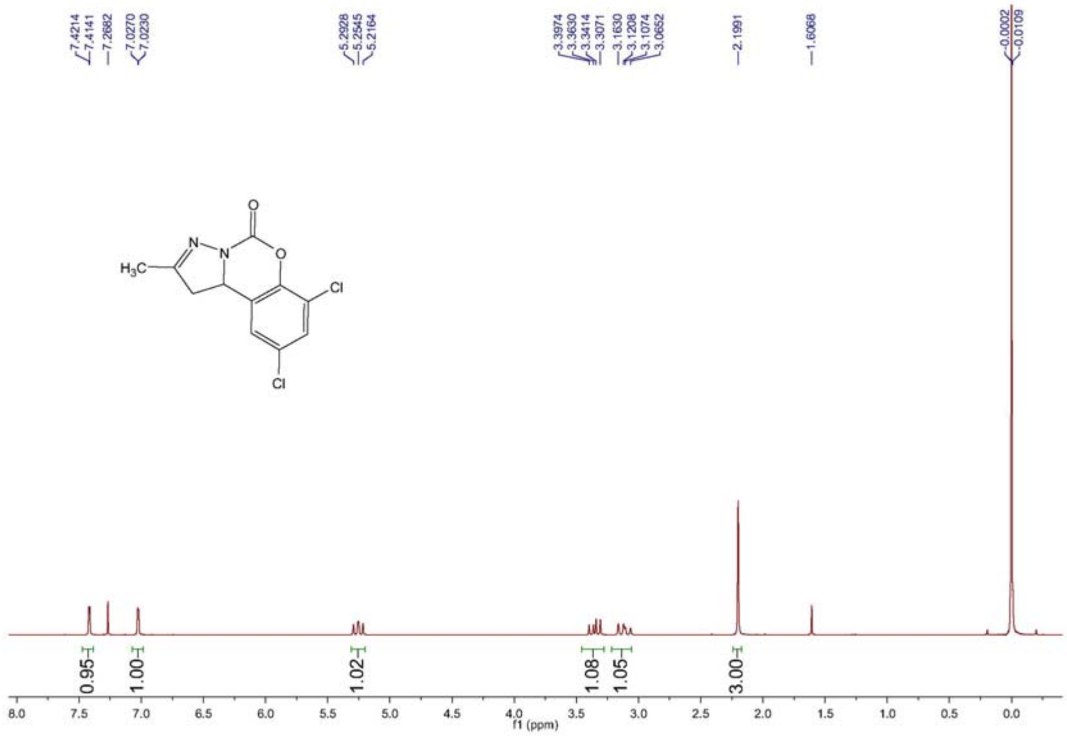
7.4214
7.4141
7.2682
7.0270
7.0230
5.2928
5.2545
5.2164
3.3974
3.3630
3.3414
3.3071
3.1630
3.1208
3.1074
3.0652
2.1991
1.6068
0.0002
-0.0109
O
N
N
O
H3C
Cl
Cl
0.95
1.00
1.02
1.08
1.05
3.00
f1 (ppm)

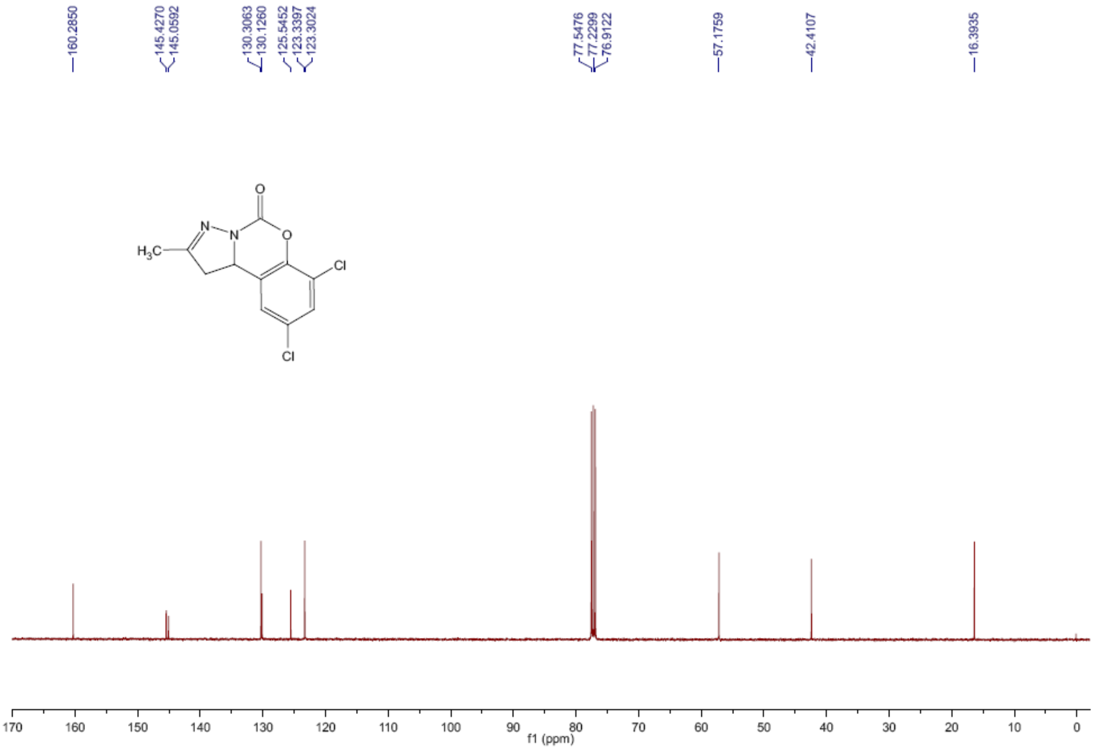
160.2850
145.4270
145.0592
130.3063
130.1260
125.5452
123.3397
123.3024
77.5476
77.2299
76.9122
57.1759
42.4107
16.3935
O
N
N
O
H3C
Cl
Cl
f1 (ppm)

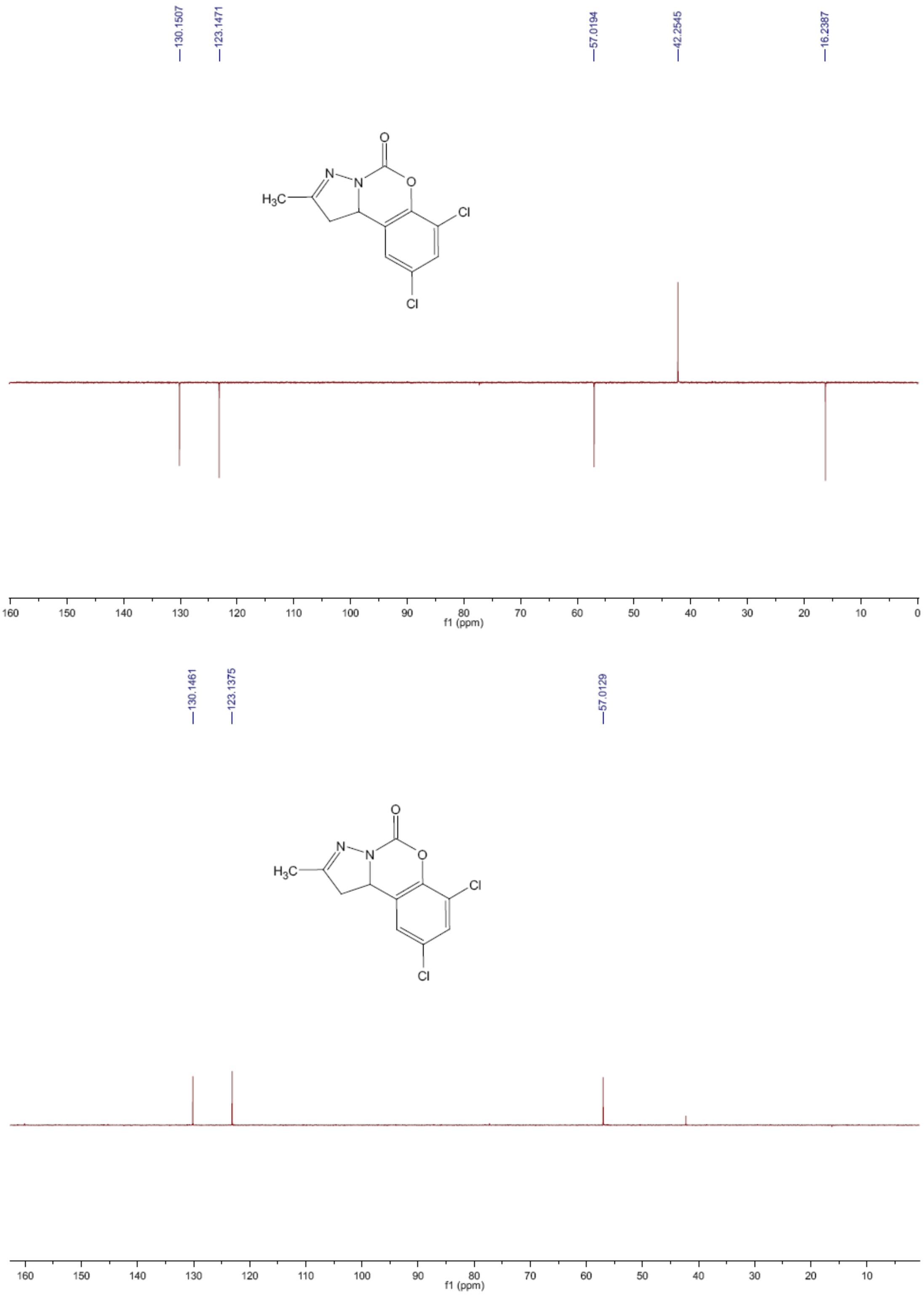


**Figure S7.** $^{1}$H-NMR, $^{13}$C-NMR and DEPT spectra of compound **6a**.

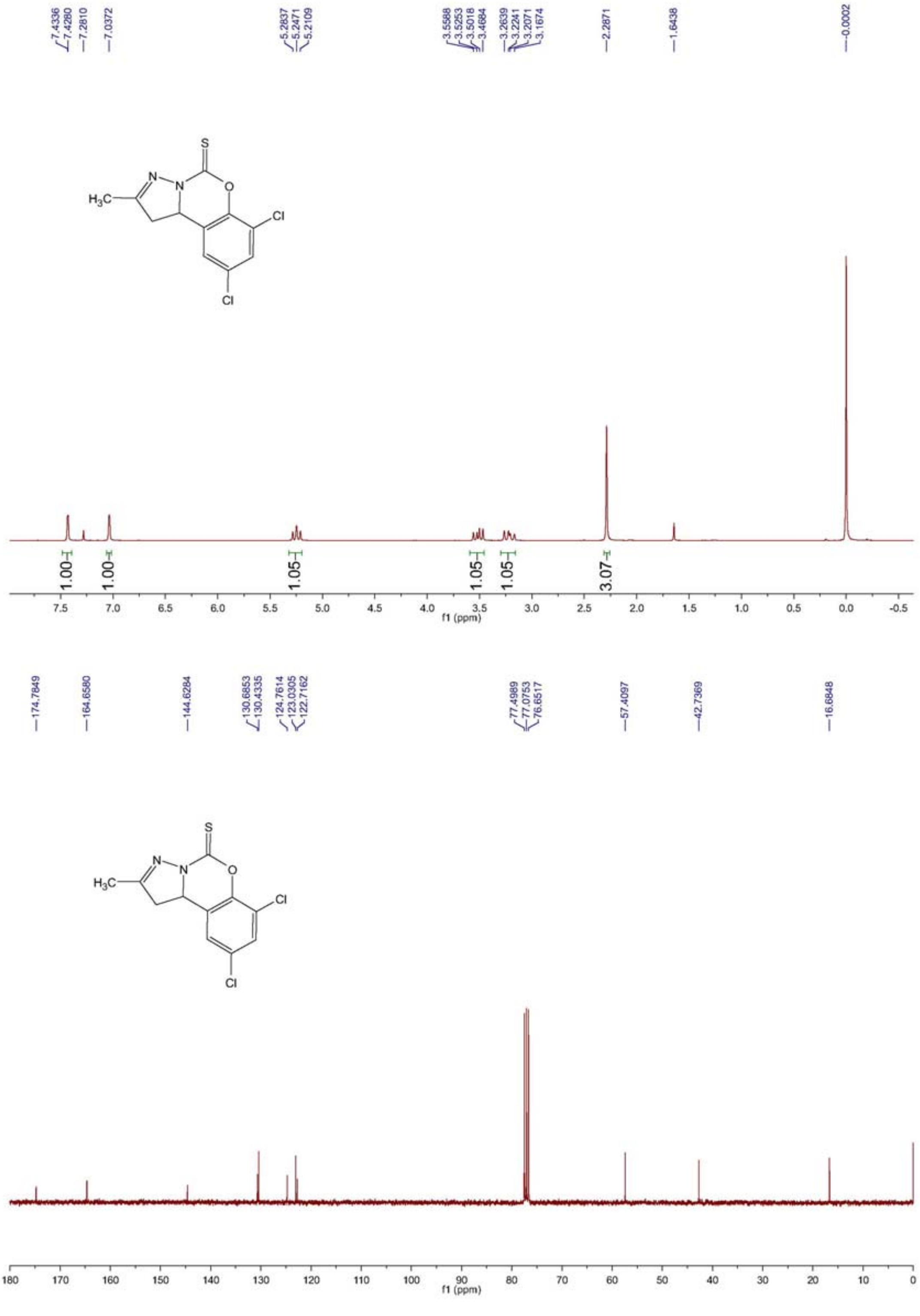


**Figure S8.** $^{1}$H-NMR and $^{13}$C-NMR spectra of compound **6b**.

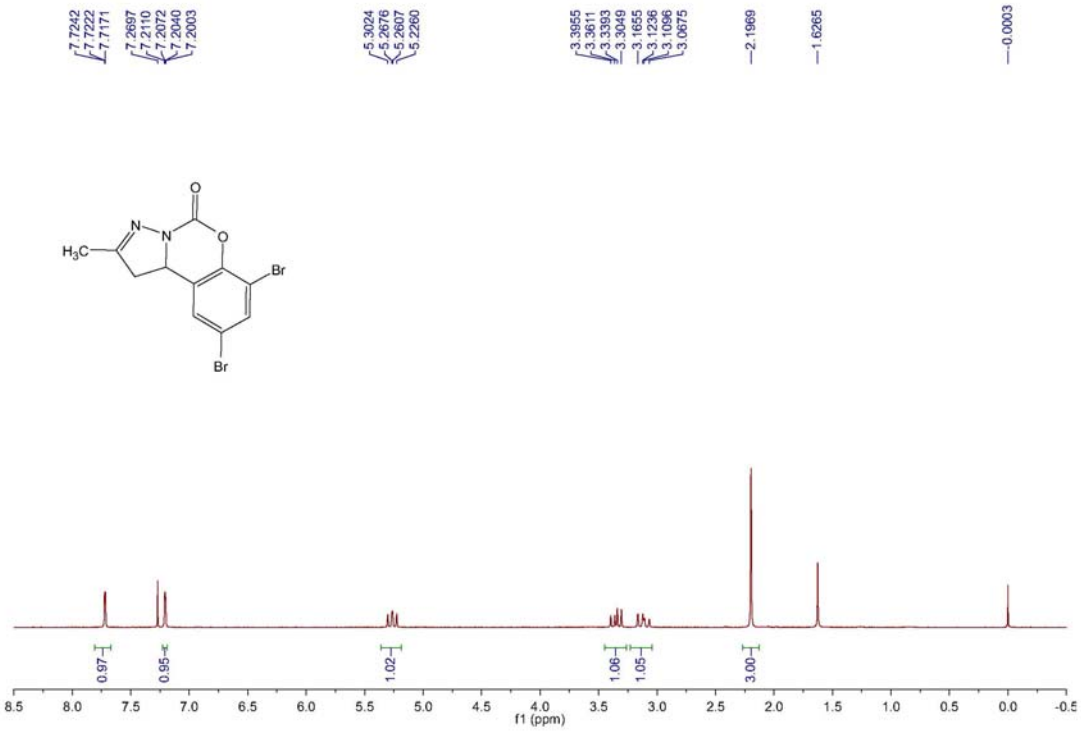

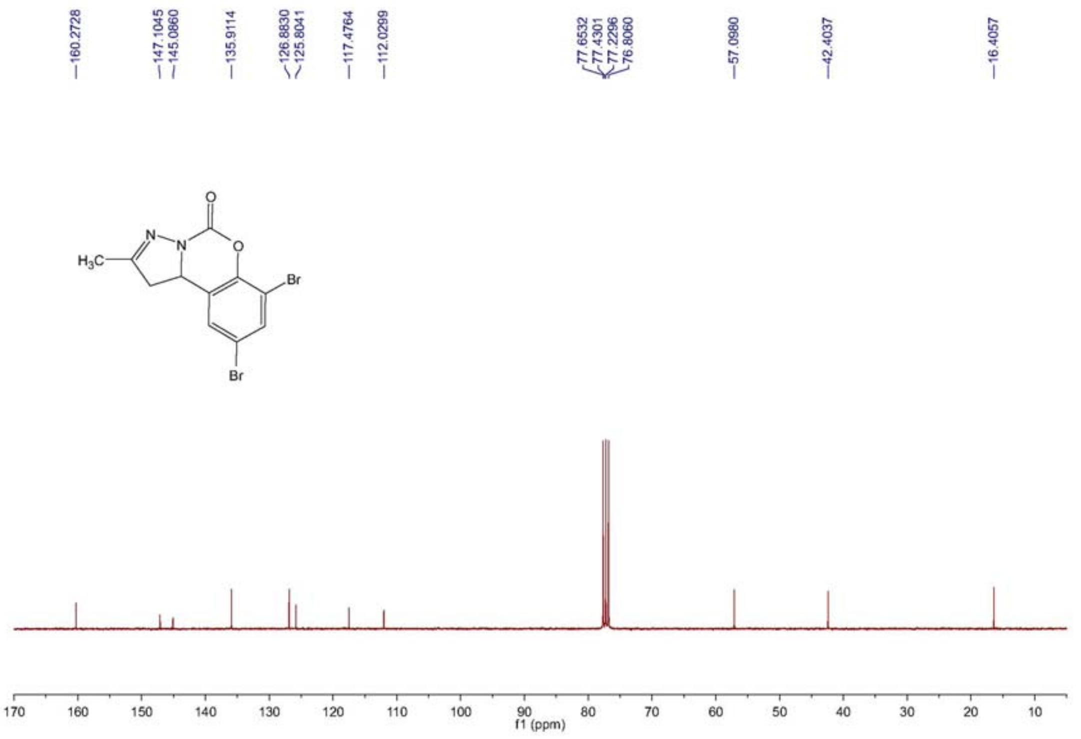

**Figure S9.** $^{1}H$-NMR and $^{13}C$-NMR spectra of compound **6c**.

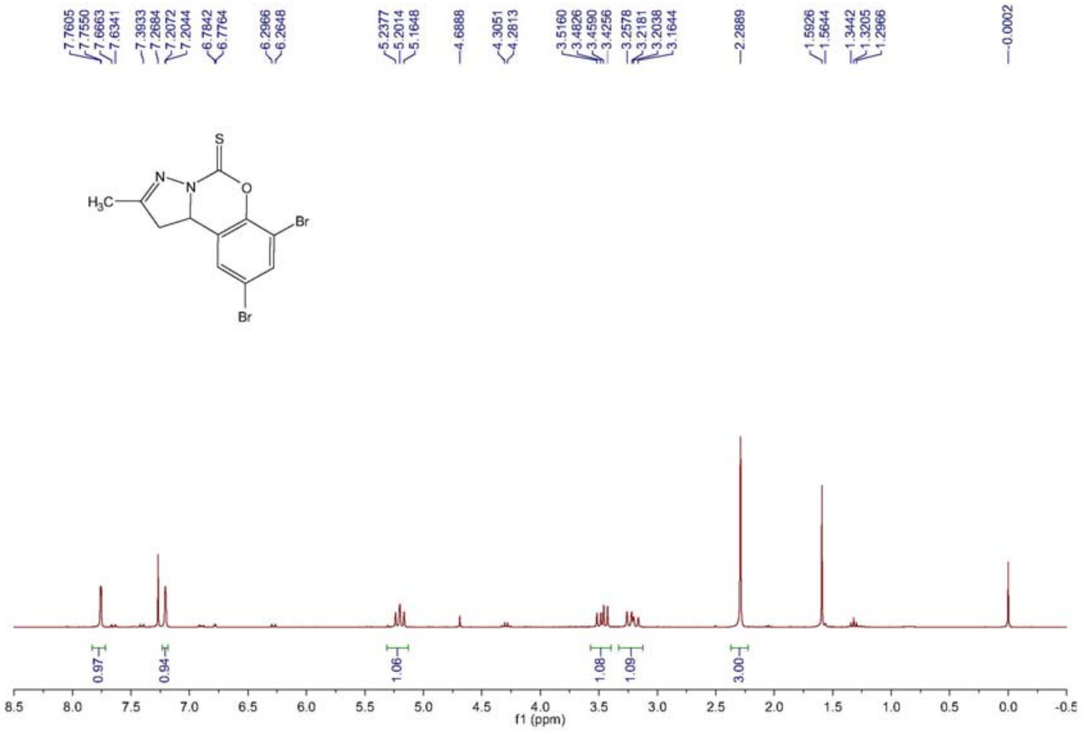

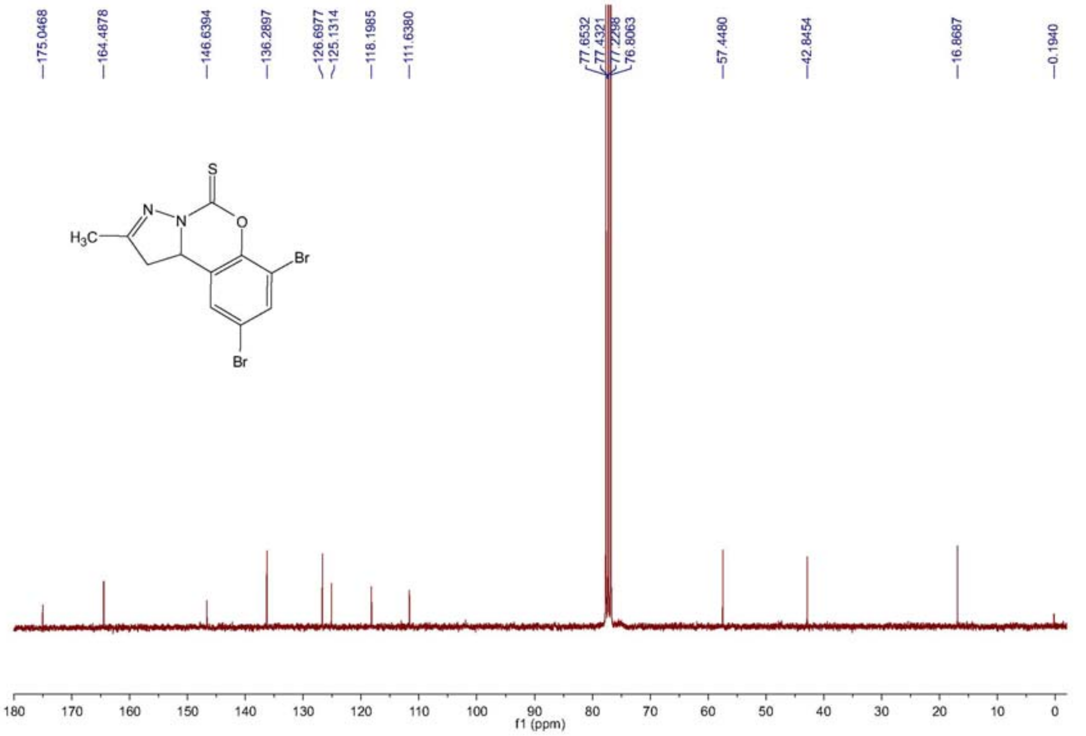

**Figure S10.** $^{1}$H-NMR and $^{13}$C-NMR spectra of compound **6d**.

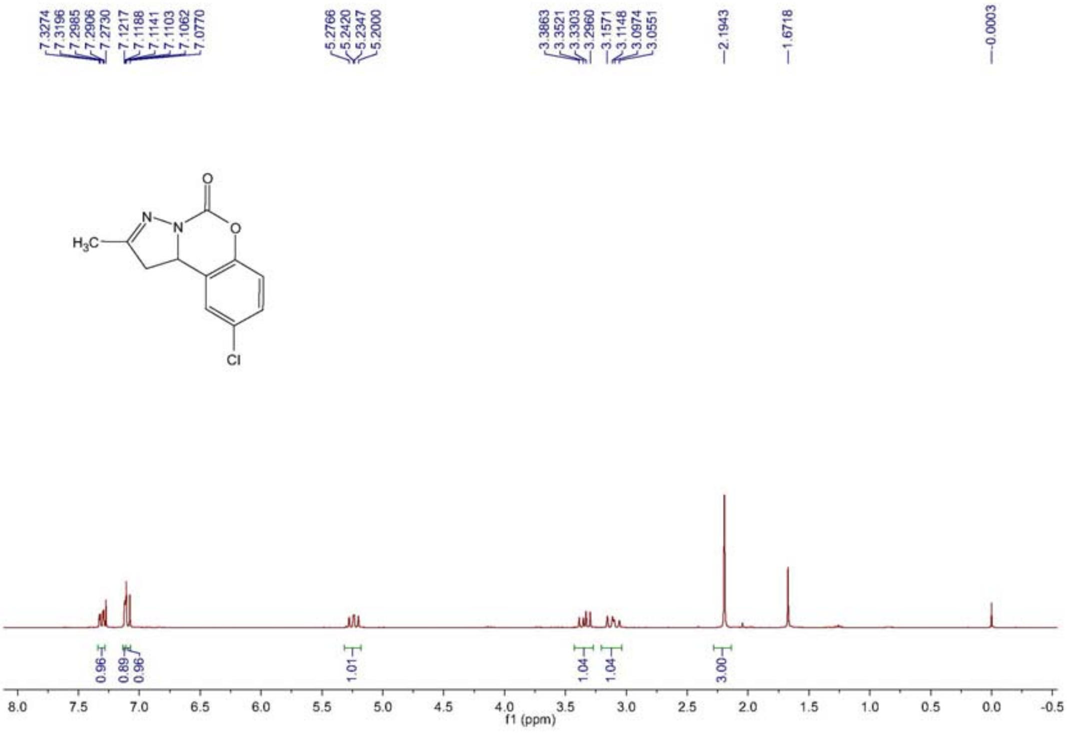

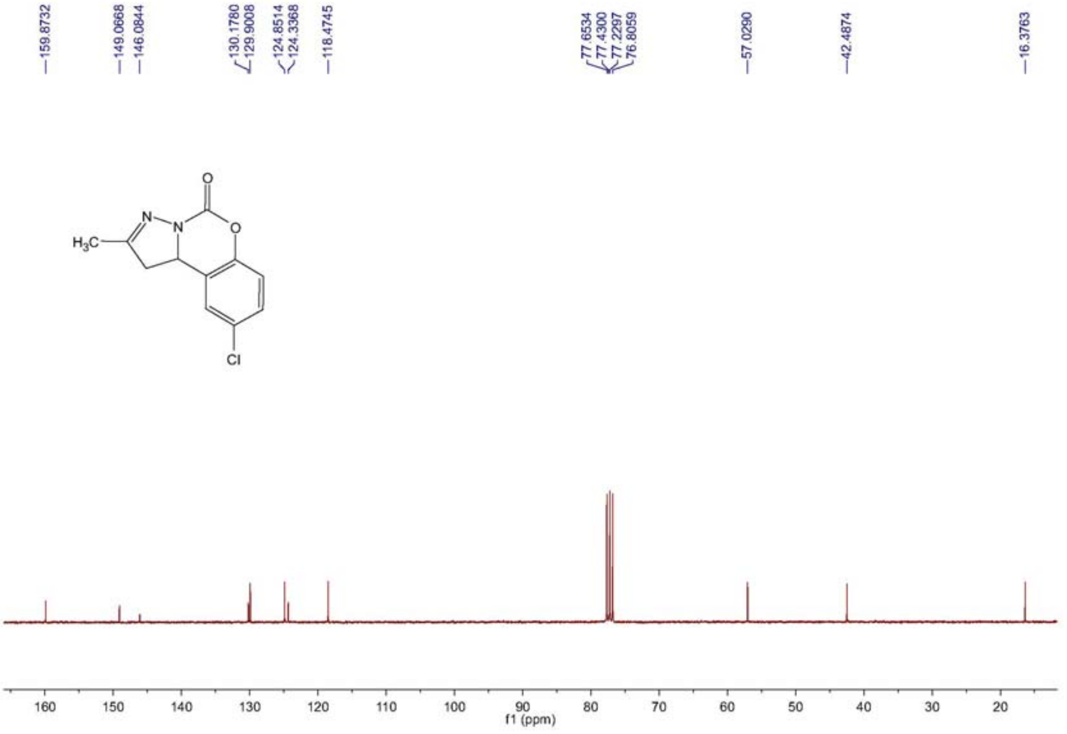

**Figure S11.** $^{1}$H-NMR and $^{13}$C-NMR spectra of compound **6e**.

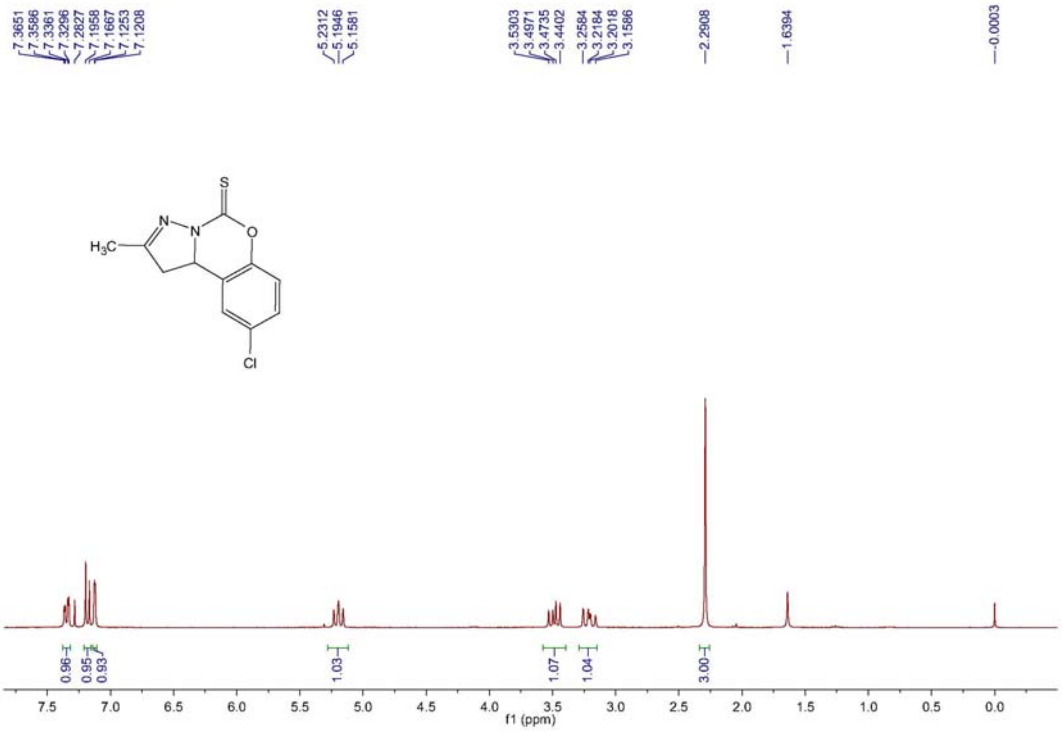


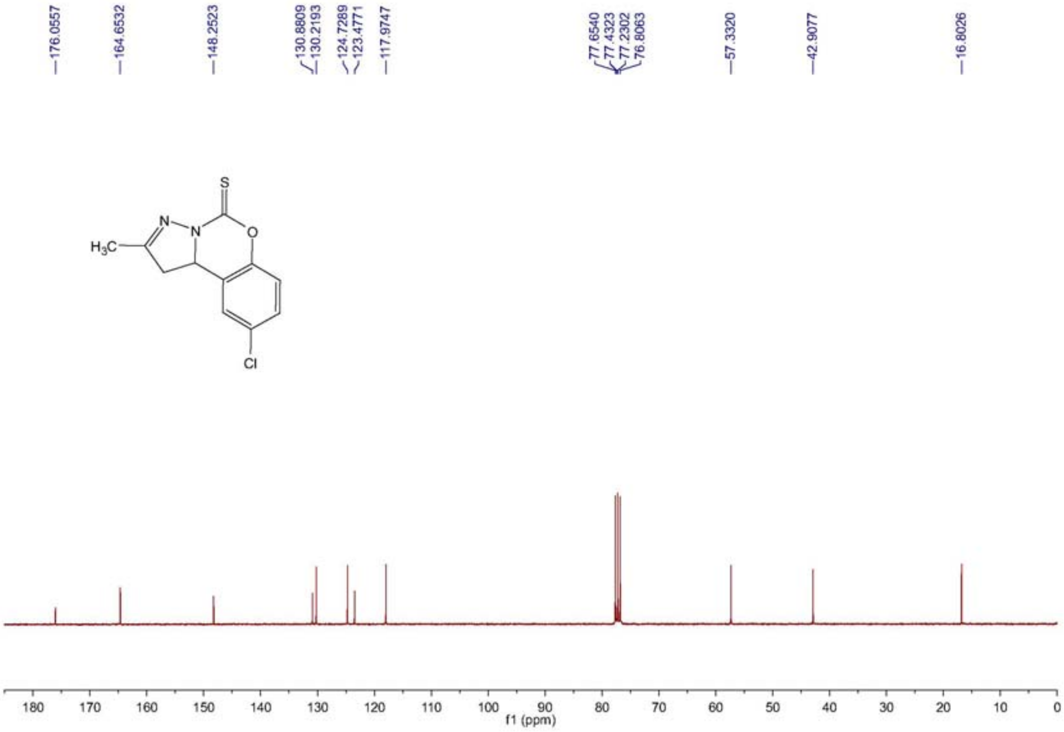


**Figure S12.** $^{1}$H-NMR and $^{13}$C-NMR spectra of compound **6f**.

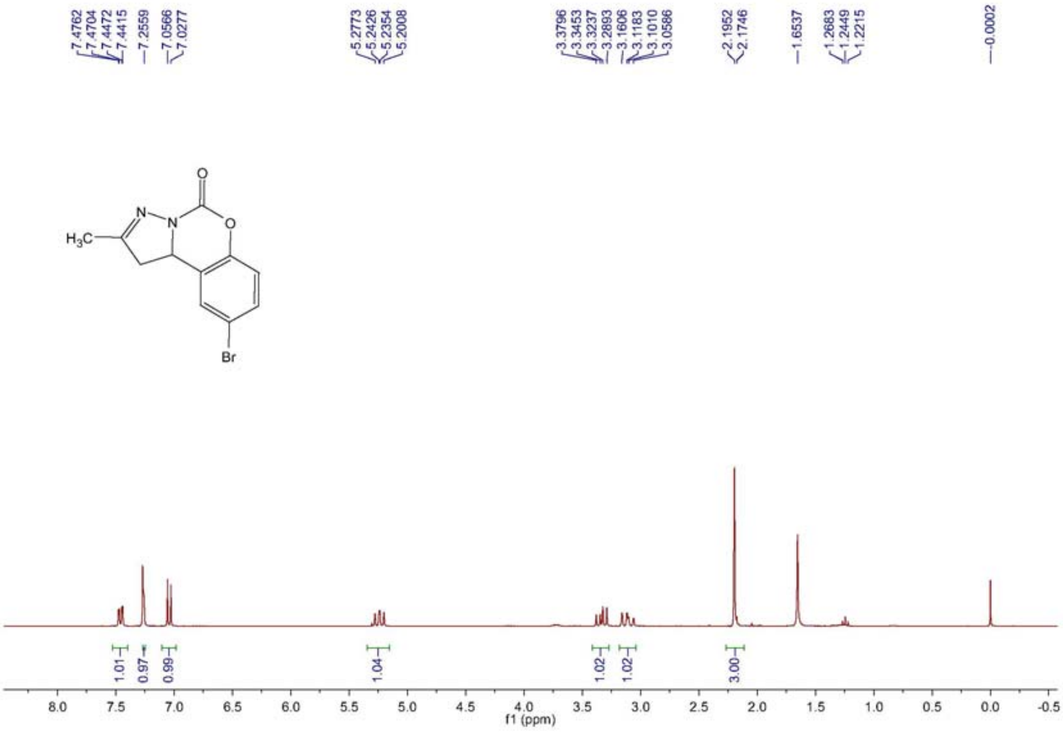


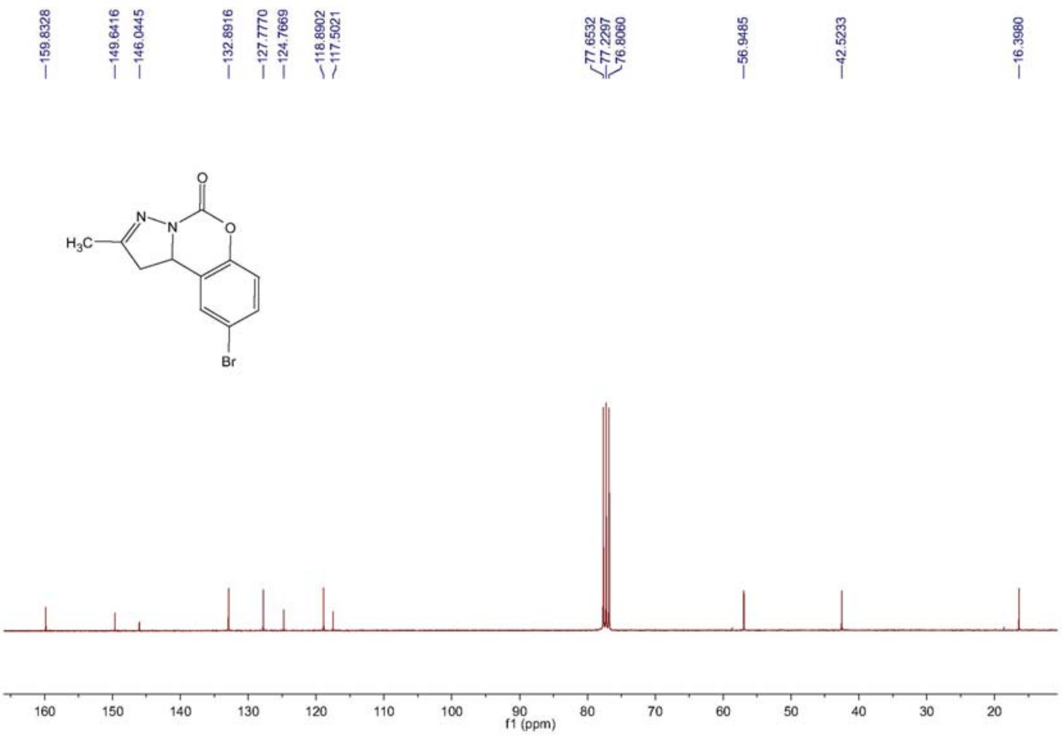


**Figure S13.** $^{1}$H-NMR and $^{13}$C-NMR spectra of compound **6g**.

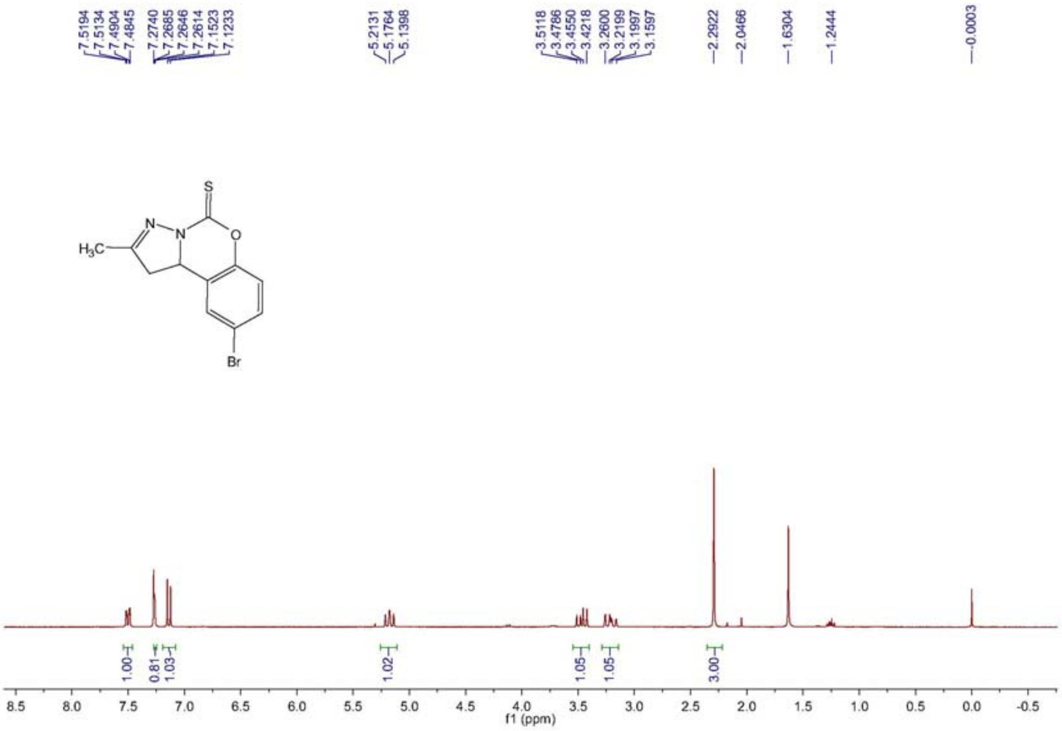


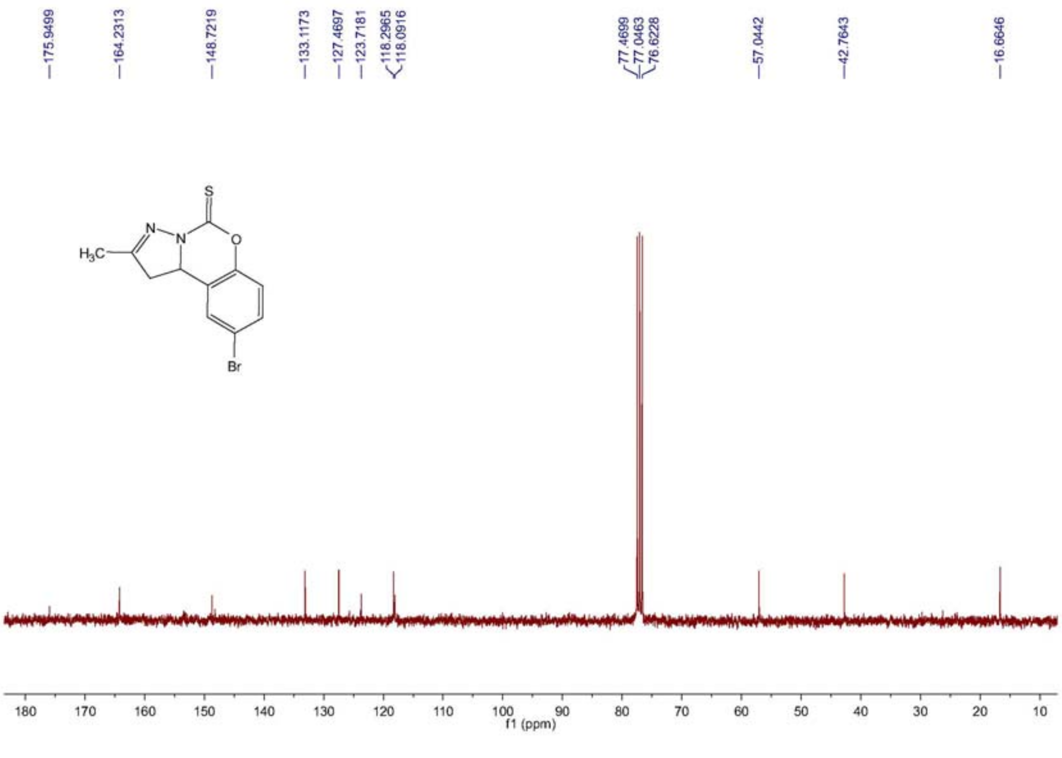


**Figure S14.** $^{1}$H-NMR and $^{13}$C-NMR spectra of compound **6h**.

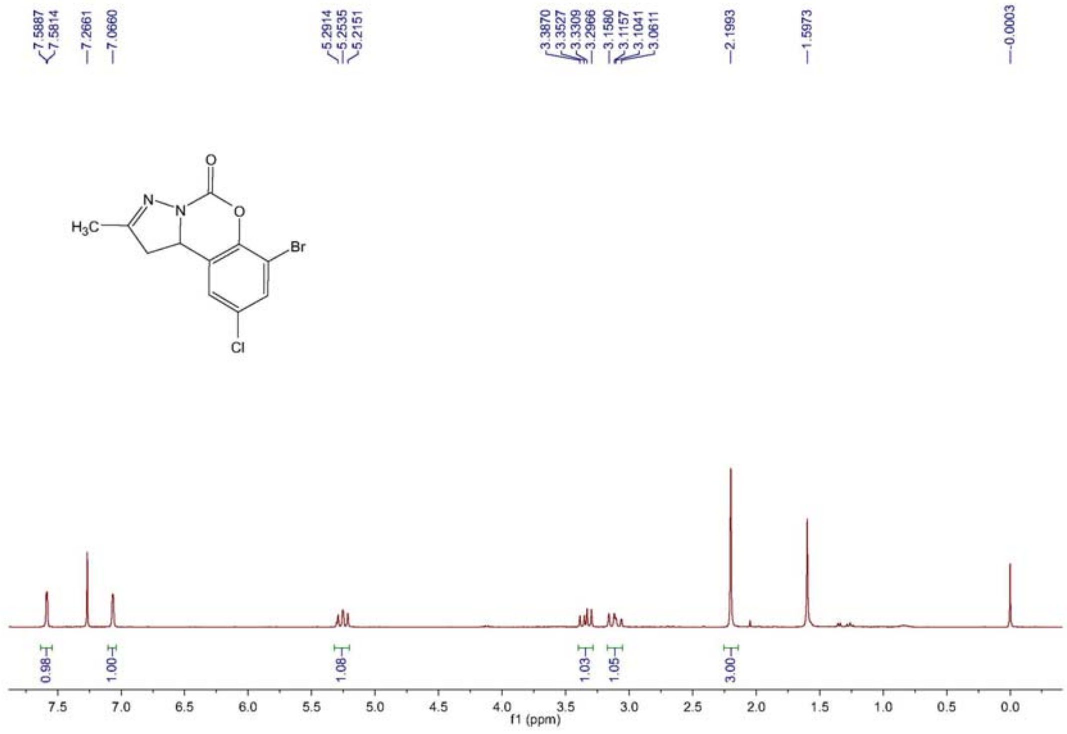


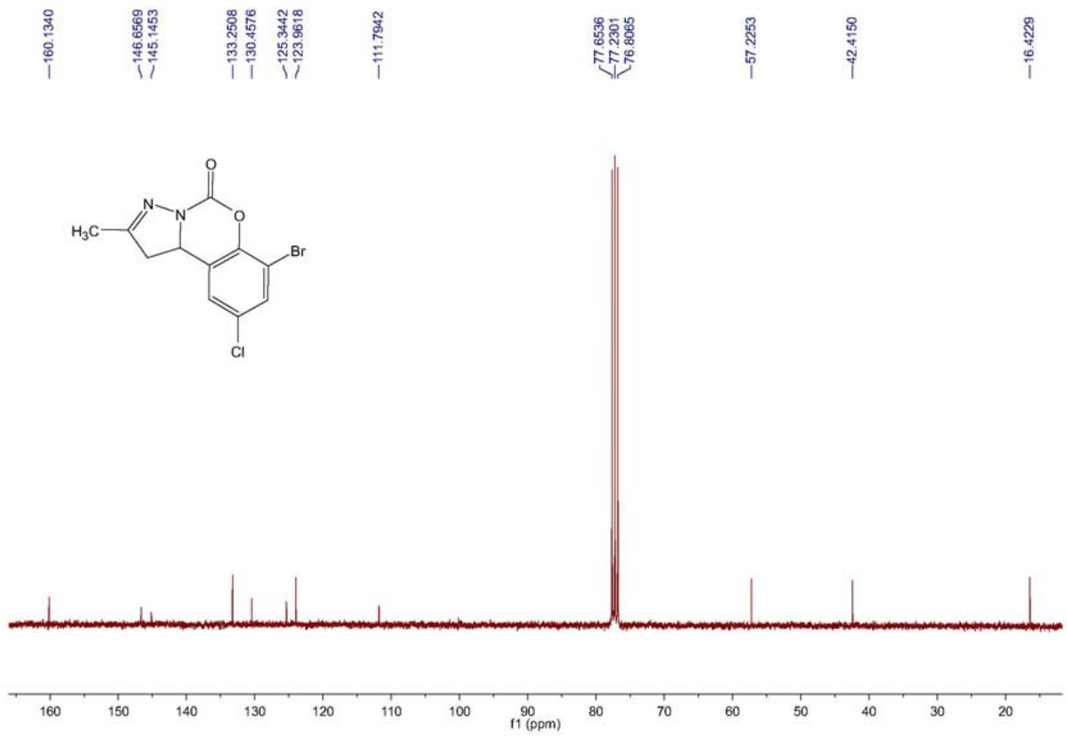


**Figure S15.** $^{1}$H-NMR and $^{13}$C-NMR spectra of compound **6i**.

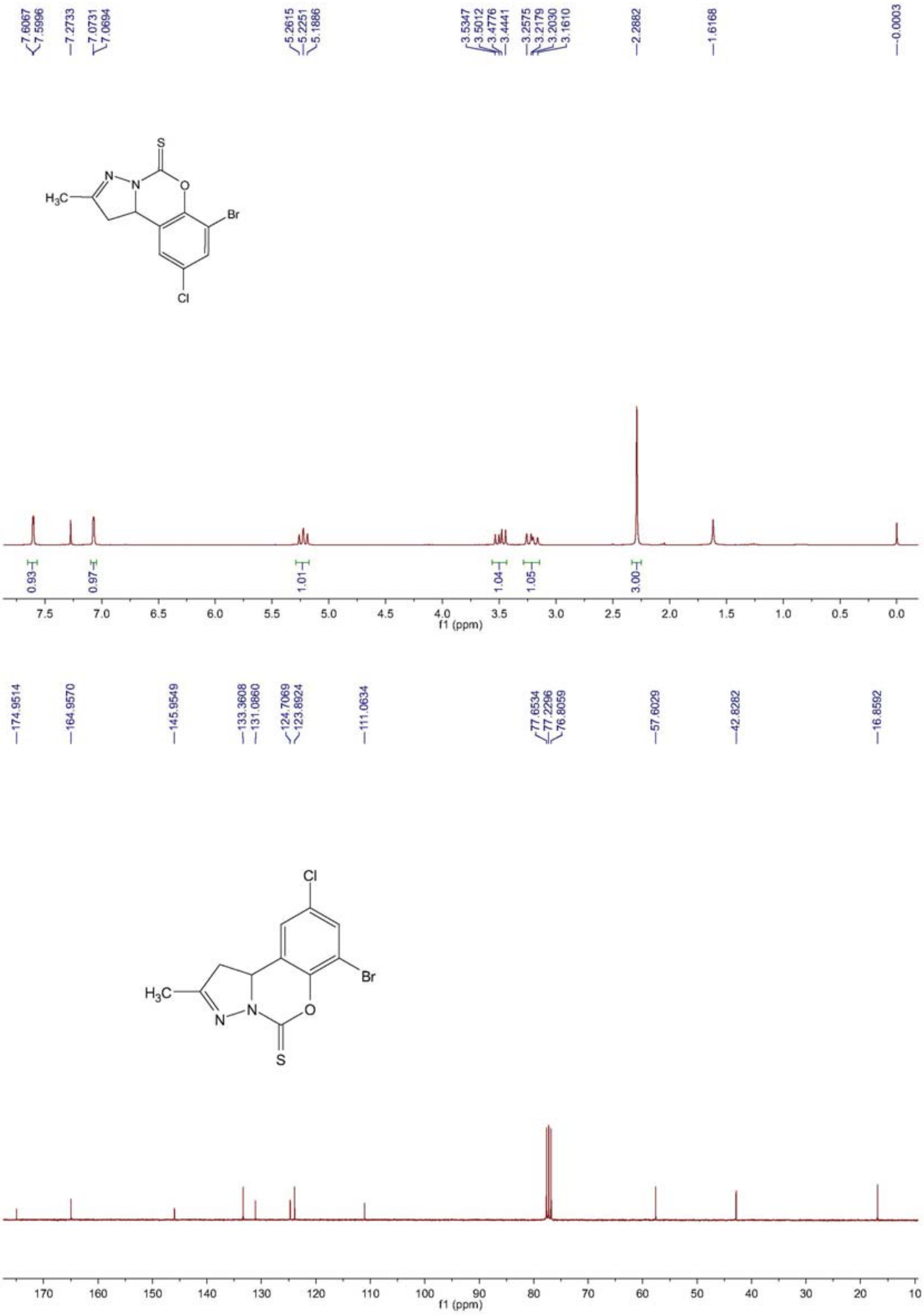


**Figure S16.** $^{1}$H-NMR and $^{13}$C-NMR spectra of compound **6j**.

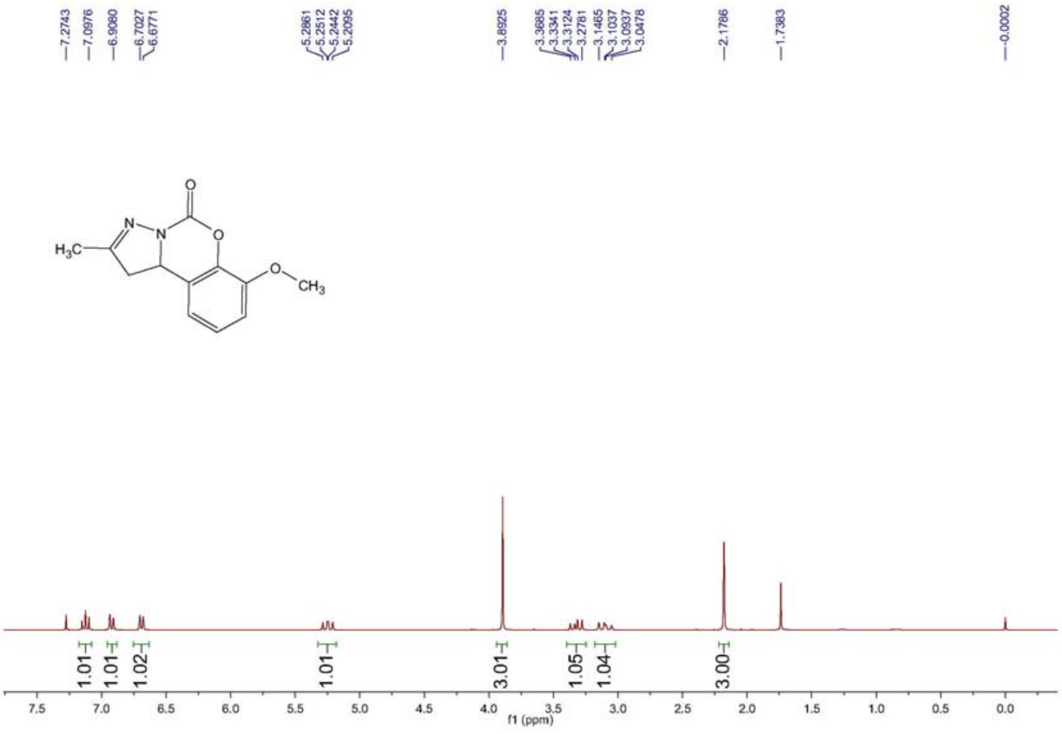


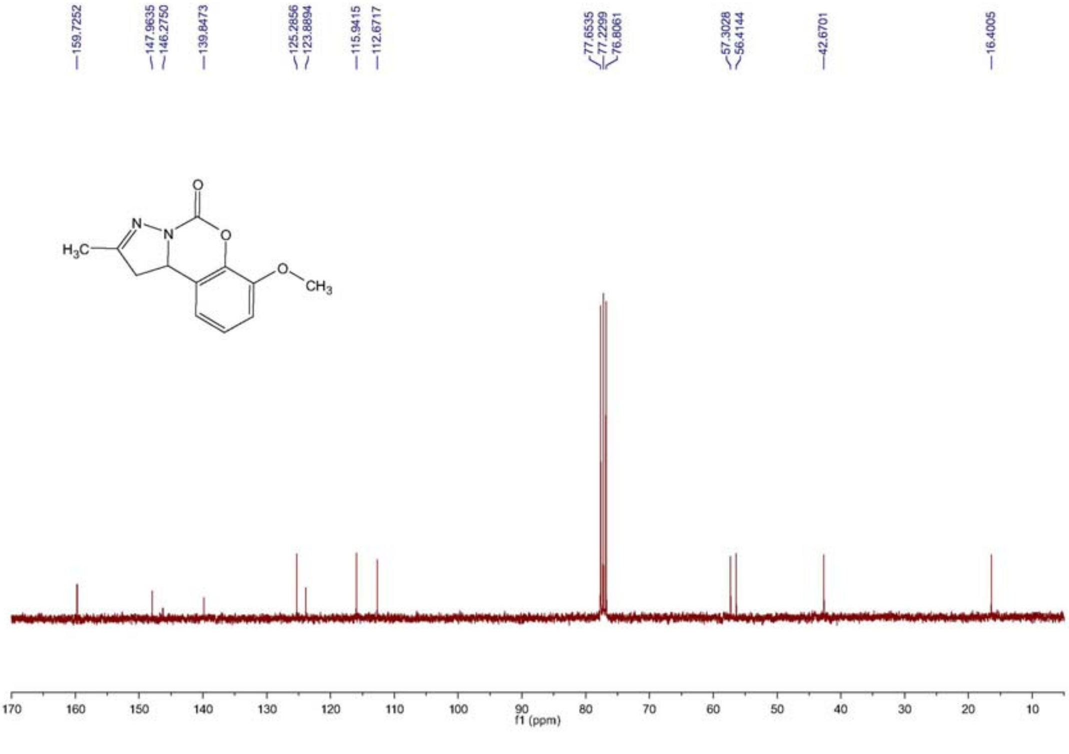


**Figure S17.** $^{1}$H-NMR and $^{13}$C-NMR spectra of compound **6k**.

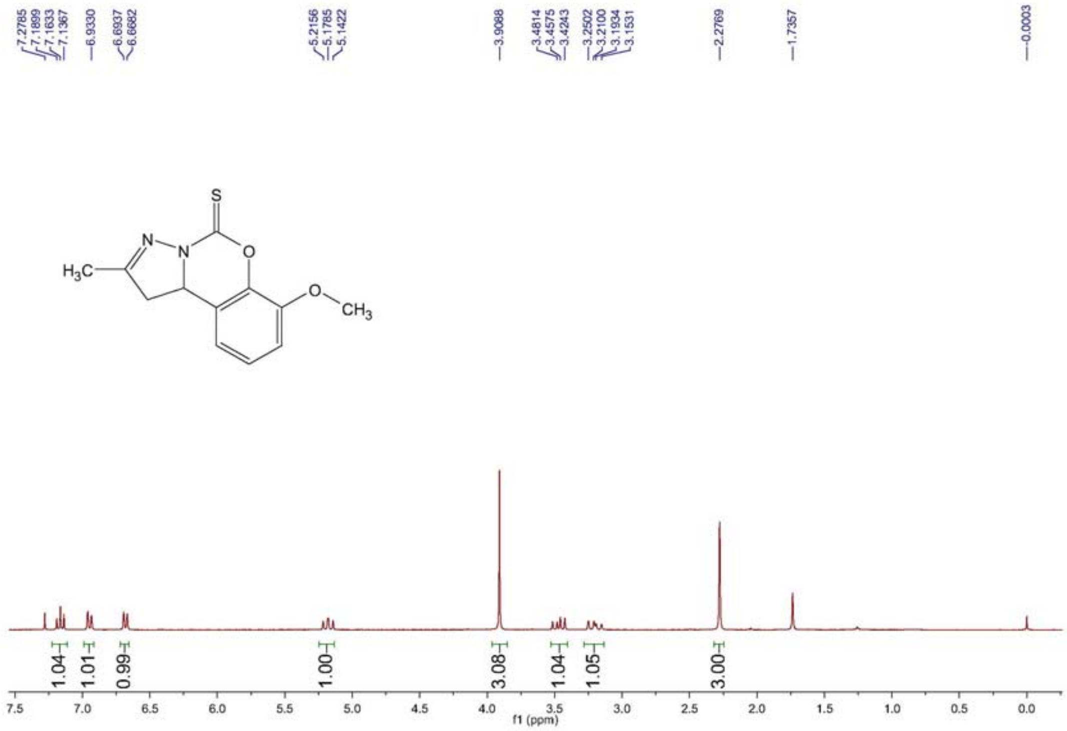


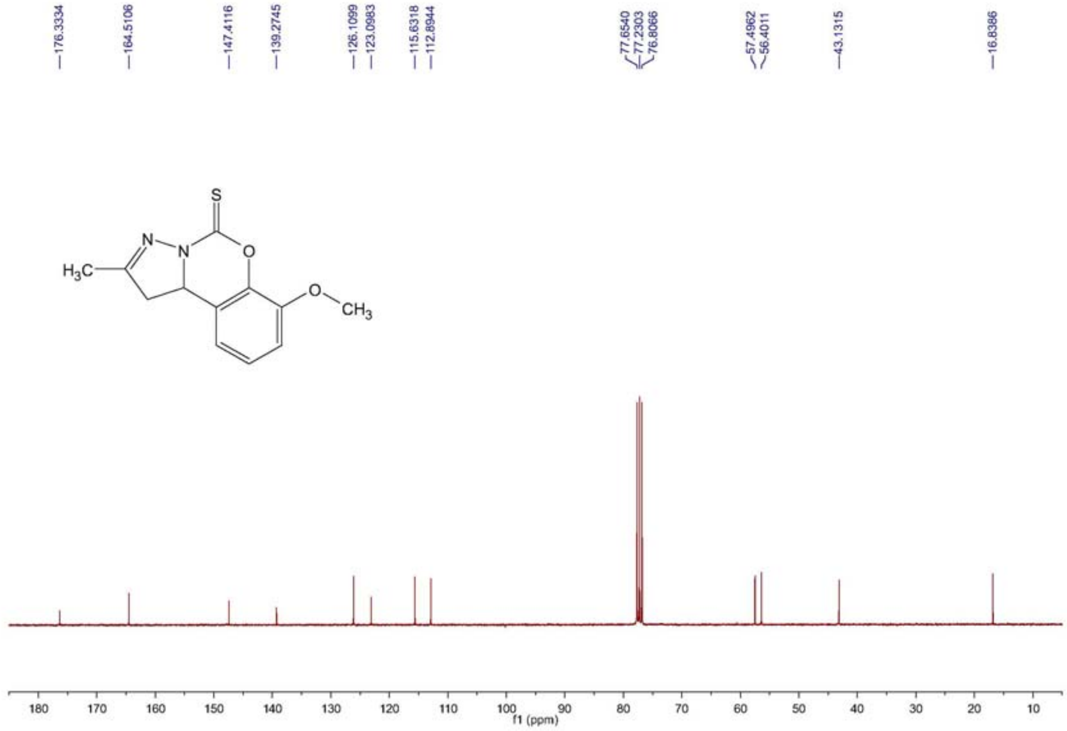


**Figure S18.** $^{1}$H-NMR and $^{13}$C-NMR spectra of compound **6l**.

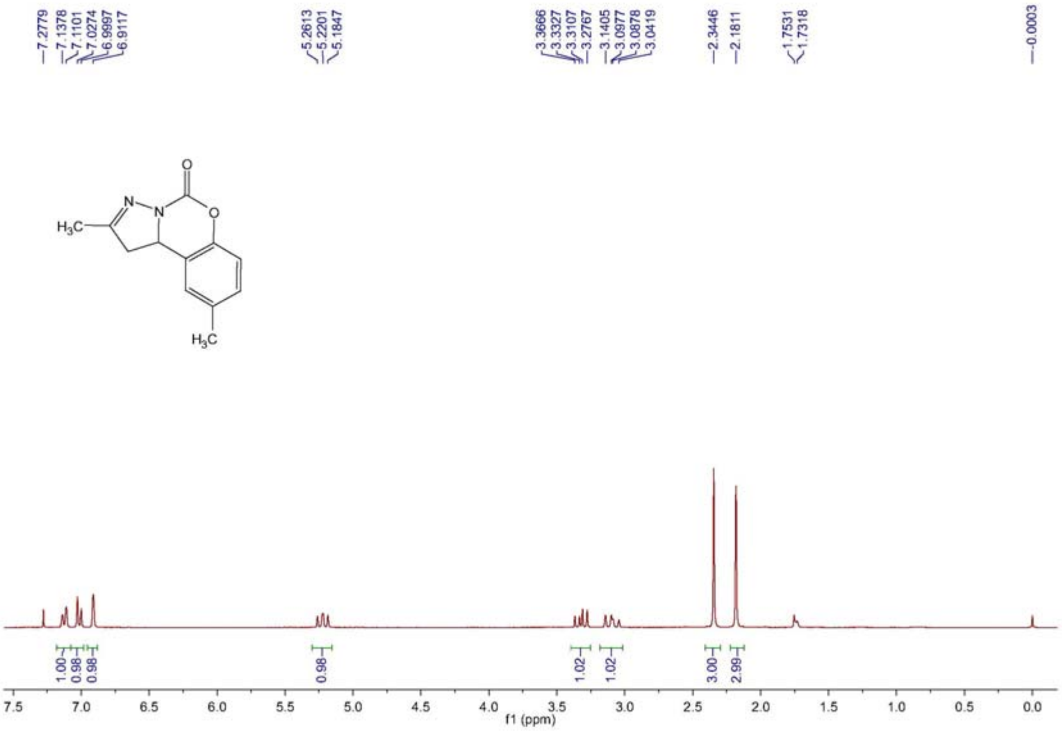


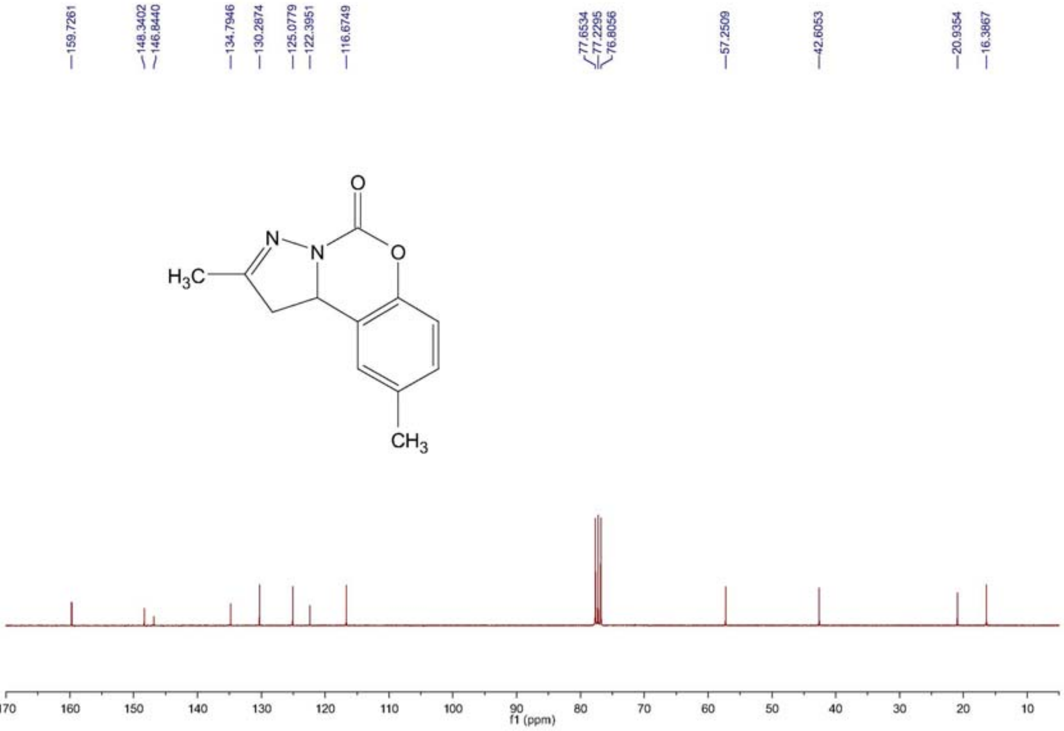


**Figure S19.** $^{1}$H-NMR and $^{13}$C-NMR spectra of compound **6m**.

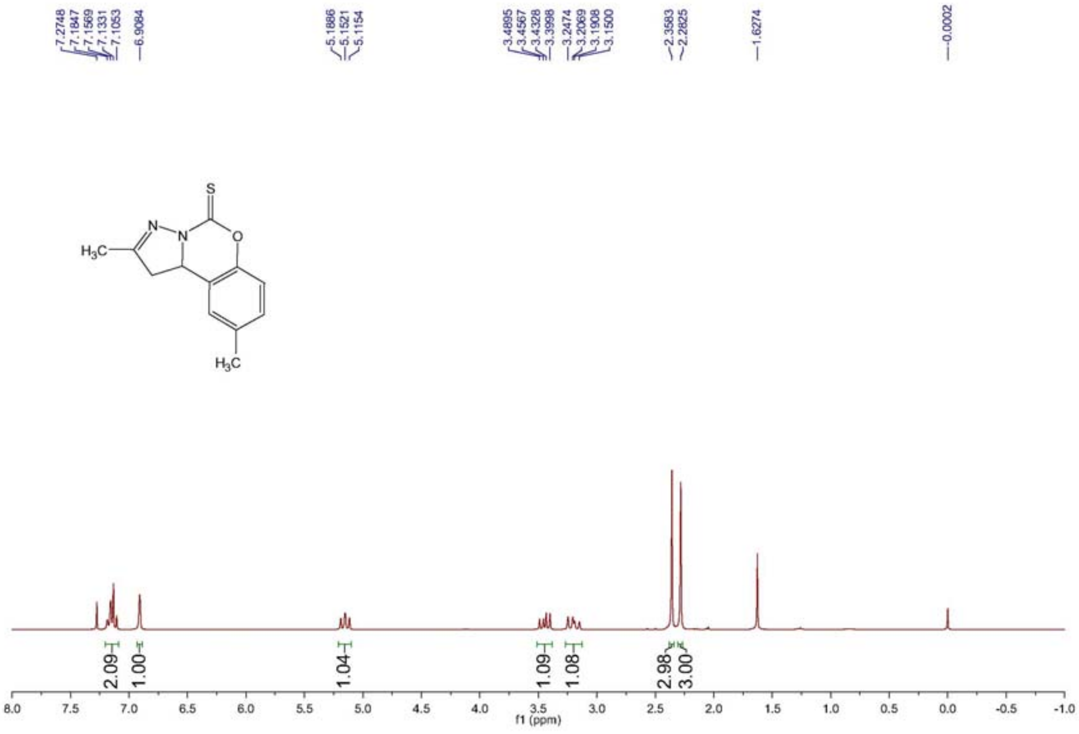

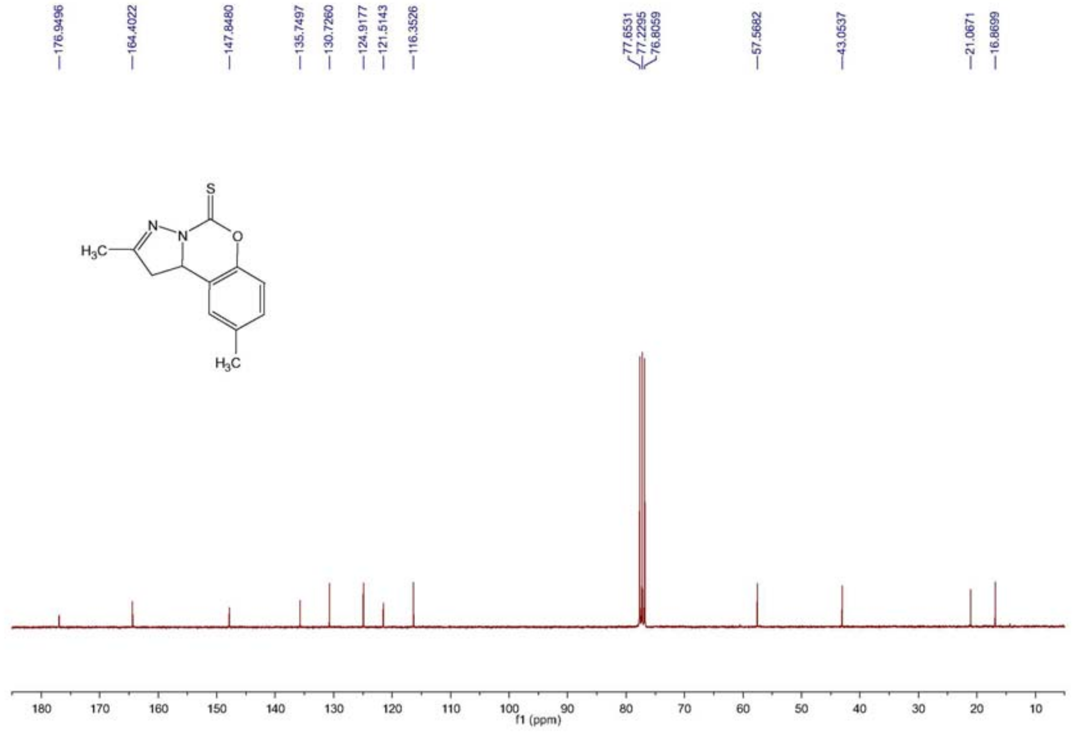

**Figure S20.** $^{1}$H-NMR and $^{13}$C-NMR spectra of compound **6n**.

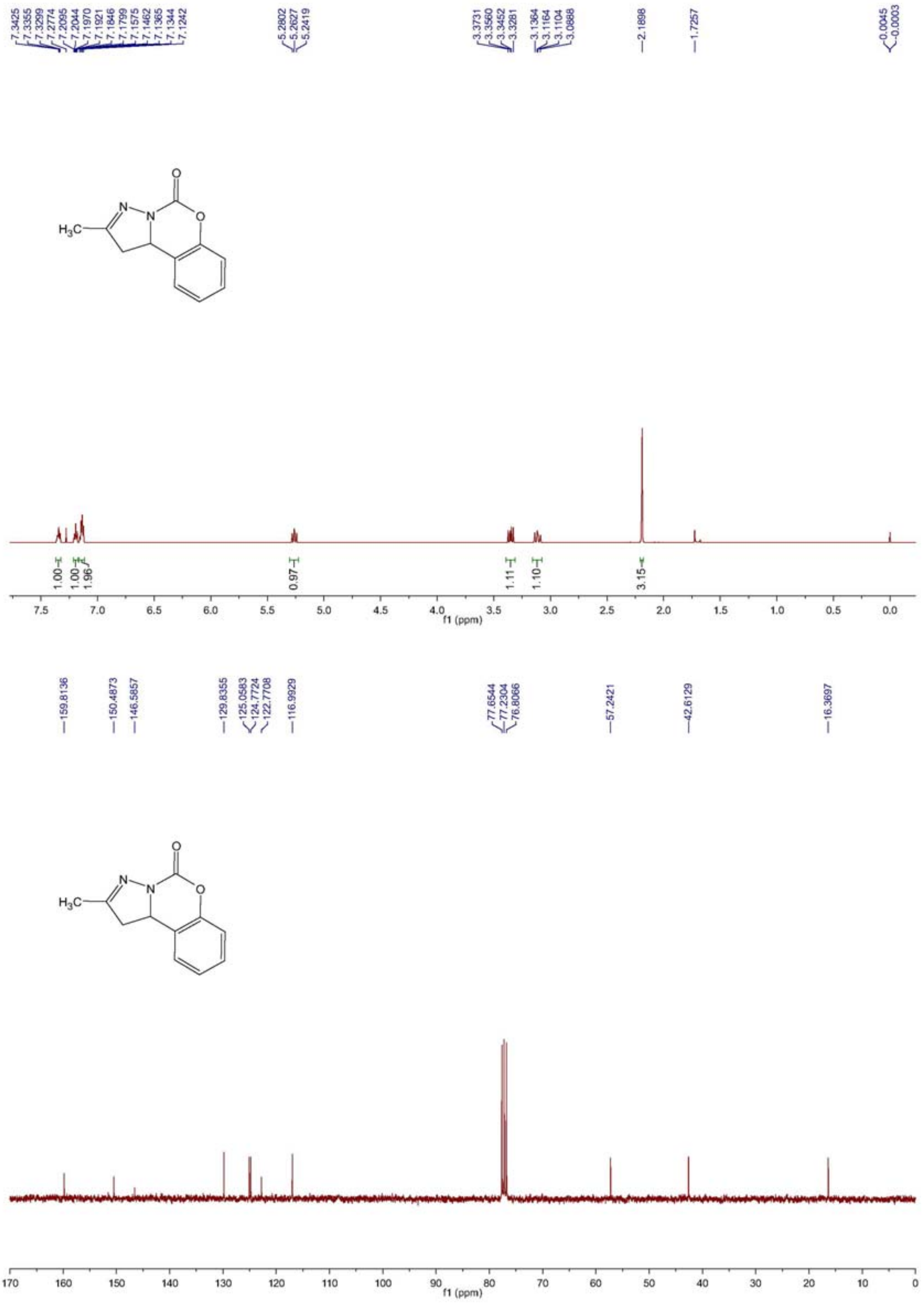


**Figure S21.** $^{1}$H-NMR and $^{13}$C-NMR spectra of compound **6o**.

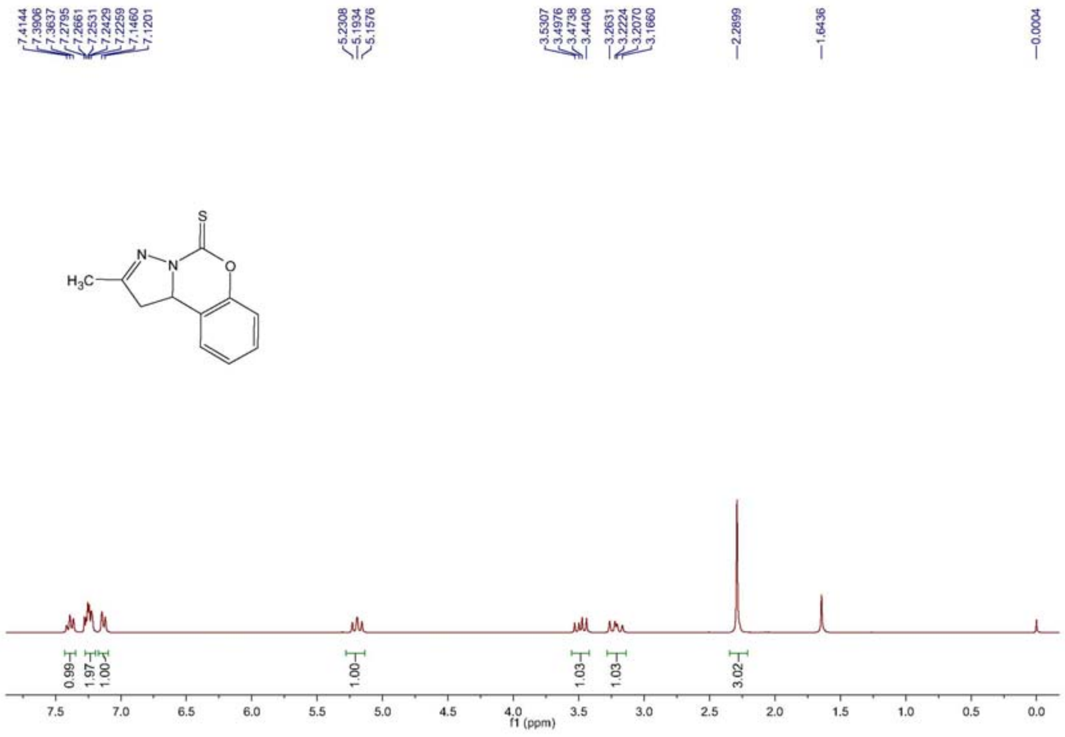
7.4144
7.3906
7.3637
7.2795
7.2661
7.2531
7.2429
7.2259
7.1460
7.1201
5.2308
5.1934
5.1576
3.5307
3.4976
3.4738
3.4408
3.2631
3.2224
3.2070
3.1660
2.2899
1.6436
0.0004
S
N
N
O
$H_3C$
0.99
1.97
1.00
1.00
1.03
1.03
3.02
7.5 7.0 6.5 6.0 5.5 5.0 4.5 4.0 3.5 3.0 2.5 2.0 1.5 1.0 0.5 0.0
f1 (ppm)

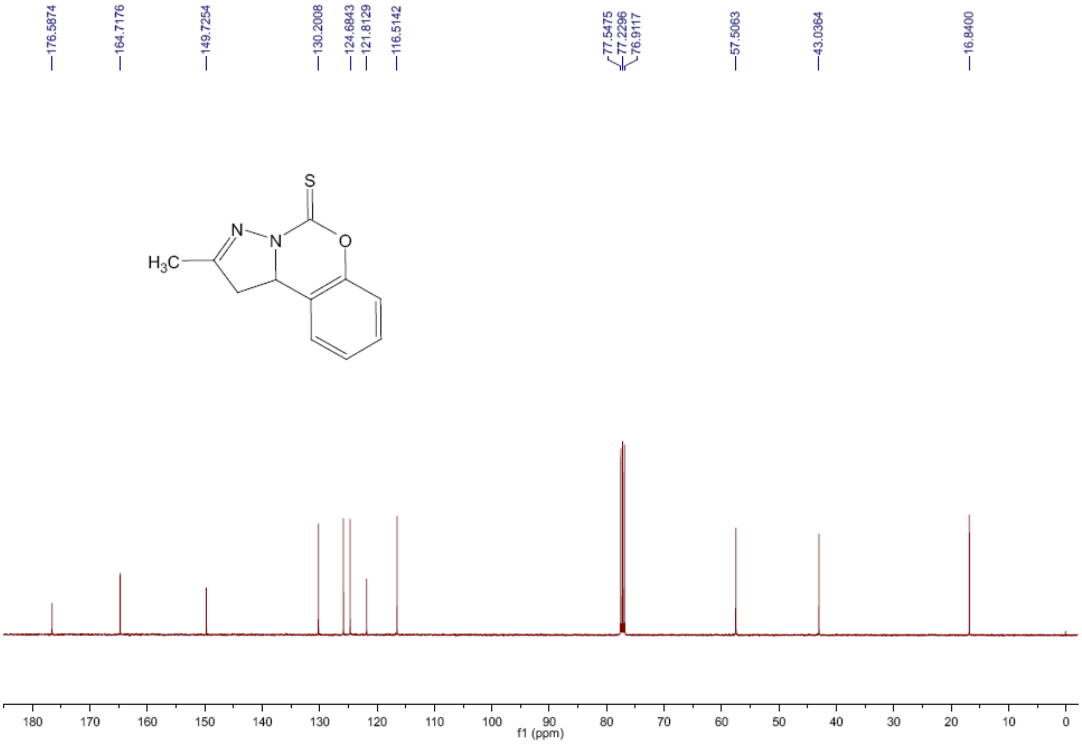
176.5874
164.7176
149.7254
130.2008
124.6843
121.8129
116.5142
77.5475
77.2296
76.9117
57.5063
43.0364
16.8400
S
N
N
O
$H_3C$
180 170 160 150 140 130 120 110 100 90 80 70 60 50 40 30 20 10 0
f1 (ppm)

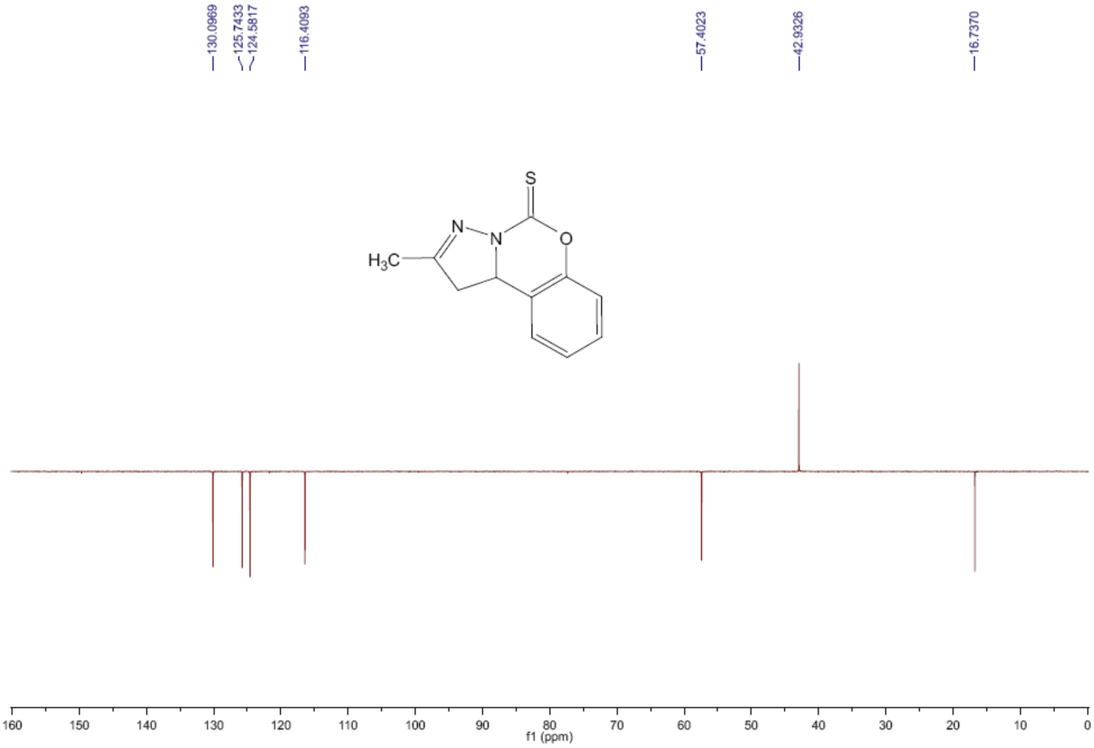


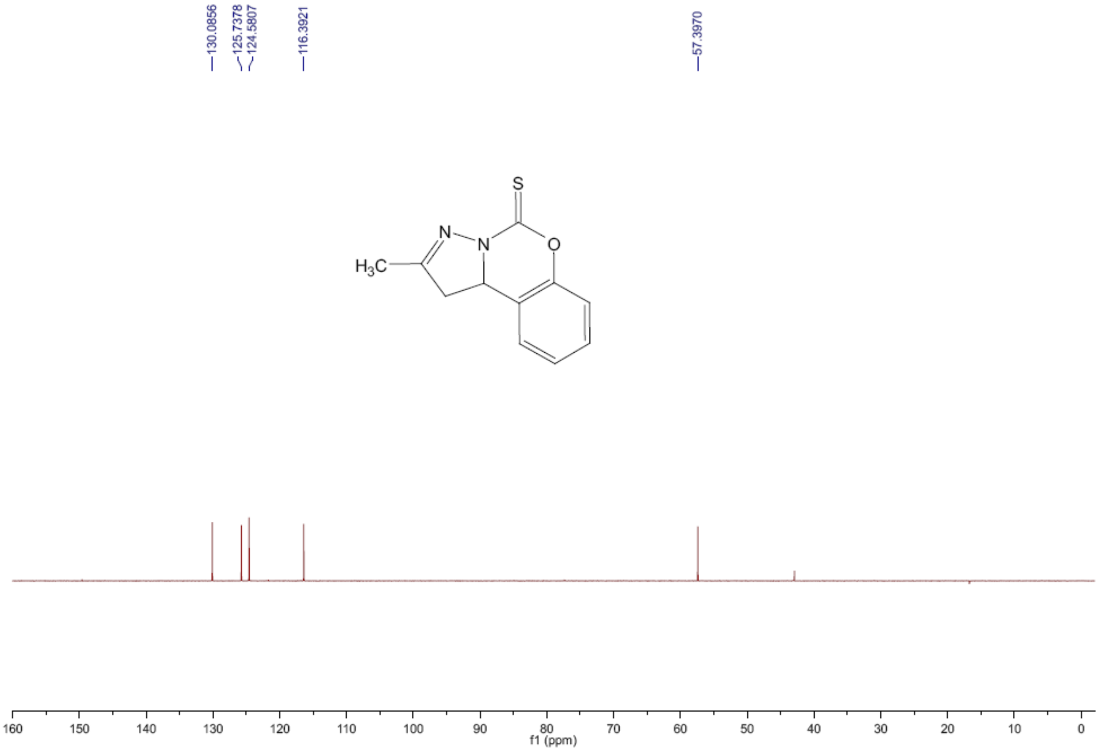


**Figure S22.** $^{1}$H-NMR, $^{13}$C-NMR and DEPT spectra of compound **6p**.

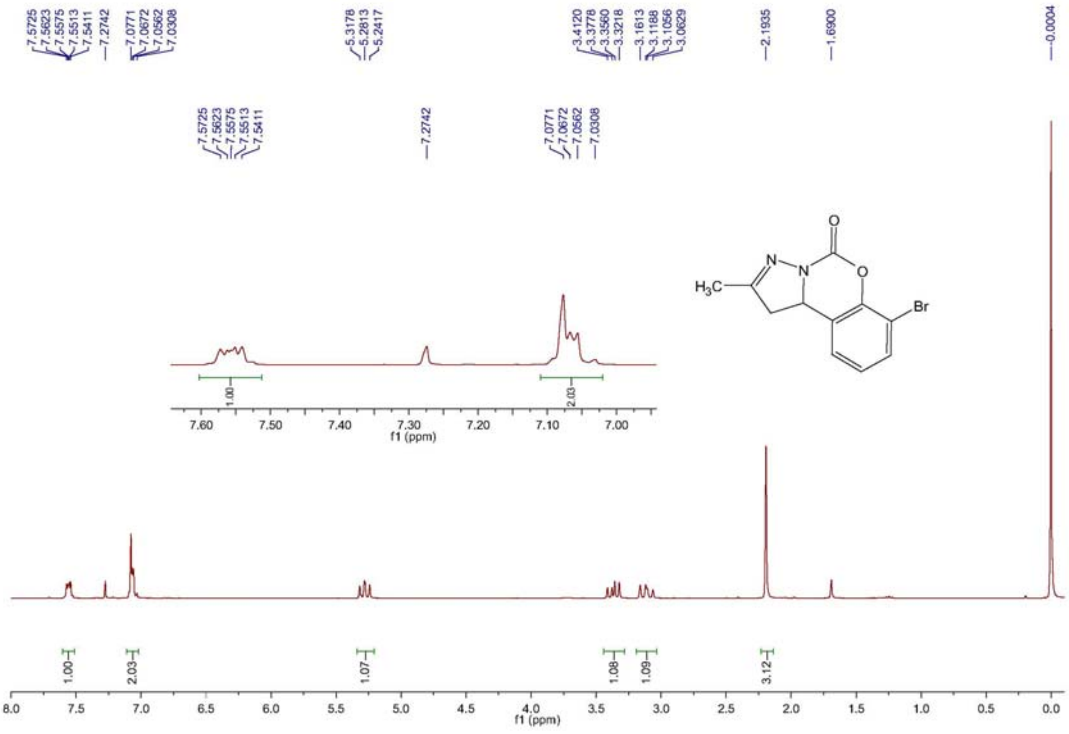


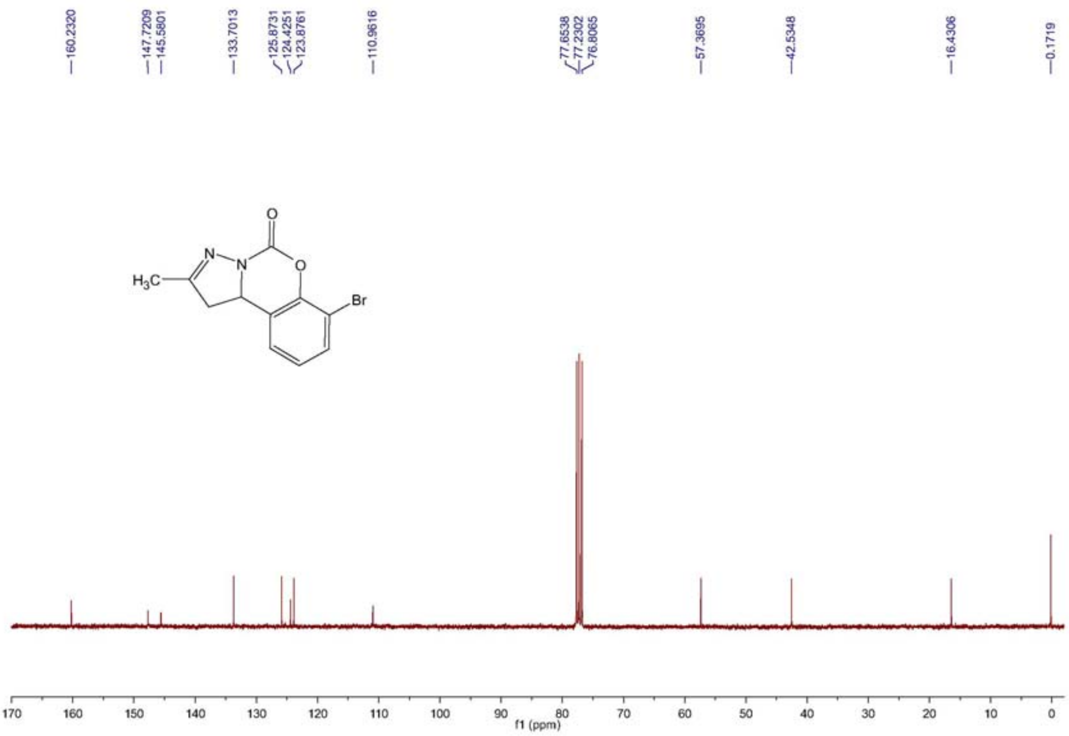


**Figure S23.** $^{1}$H-NMR and $^{13}$C-NMR spectra of compound **6q**.
